# Dispersive micro-solid-phase extraction coupled to UHPLC-Q-TOF-MS and UHPLC-QqQ-MS for suspect and target screening of illicit drugs and pharmaceuticals in wastewater

**DOI:** 10.1007/s00216-026-06520-3

**Published:** 2026-04-28

**Authors:** Madson M. Nascimento, Paulo R. R. Mesquita, Ricardo L. Cunha, André L. S. da Silva Junior, Gisele O. da Rocha, Raildo M. de Jesus, Pedro Afonso de P. Pereira, Jailson B. de Andrade

**Affiliations:** 1 Universidade SENAI CIMATEC, Av. Orlando Gomes, 1845 - Piatã, Salvador, BA 41650-010 Brazil; 2 Centro Tecnológico Agropecuário do Estado da Bahia - CETAB, Secretaria da Agricultura, Pecuária, Irrigação, Pesca e Aquicultura – SEAGRI, Av. Milton Santos, 967 - Ondina, Salvador, BA 40170-110 Brazil; 3 Laboratório de Toxicologia Forense, Instituto de Análises e Pesquisas Forenses – IAPF, Polícia Científica, São Cristóvão, SE 49100-000 Brazil; 4 Universidade Federal da Bahia, Instituto de Química, Campus de Ondina, Salvador, BA 40170-115 Brazil; 5 Instituto Nacional de Ciência e Tecnologia em Energia e Ambiente - INCT, Universidade Federal da Bahia, Salvador, BA 40170-115 Brazil; 6 Universidade Estadual de Santa Cruz, Campus Soane Nazaré de Andrade, Rod. Jorge Amado, Km 16 - Salobrinho, Ilhéus, BA 45662-900 Brazil

**Keywords:** Illicit drugs, New psychoactive substances, Environmental pollution, D-µ-SPE, Mass spectrometry

## Abstract

The increasing global consumption of pharmaceuticals and illicit drugs, particularly new psychoactive substances, has resulted in their continuous release into aquatic environments, raising concerns regarding potential adverse effects on ecosystems and human health. In this study, a dispersive micro-solid-phase extraction using customized extraction devices was developed in combination with liquid chromatography–high-resolution mass spectrometry for target and suspect screening, followed by sensitive quantification by liquid chromatography–tandem mass spectrometry. The extraction procedure was optimized using experimental design. Optimal conditions comprised 50 mg of Oasis MCX sorbent, 100 mL of sample (pH 2, containing 7.5% NaCl, w v⁻^1^), and an extraction time of 30 min. Under these conditions, limits of quantification ranged from 4.67 to 29.3 ng L⁻^1^, and extraction recoveries varied between 73.0% and 108% for most target analytes. Application to real wastewater samples included an initial suspect screening step by liquid chromatography–high resolution mass spectrometry using data-dependent acquisition and an untargeted data-mining workflow. The screening results revealed a broad range of compounds, including central nervous system stimulants, cardiovascular drugs, opioid analgesics, and sedatives. Quantification of selected target analytes was subsequently performed by liquid chromatography–tandem mass spectrometry. Among the investigated illicit drugs, THC and cocaine were the predominant compounds detected, whereas fentanyl was present at trace levels (6.17–14.4 ng L⁻^1^). The proposed workflow enables robust, sensitive monitoring of illicit drugs and pharmaceuticals in wastewater for epidemiological and environmental surveillance applications.

**Graphical abstract:**

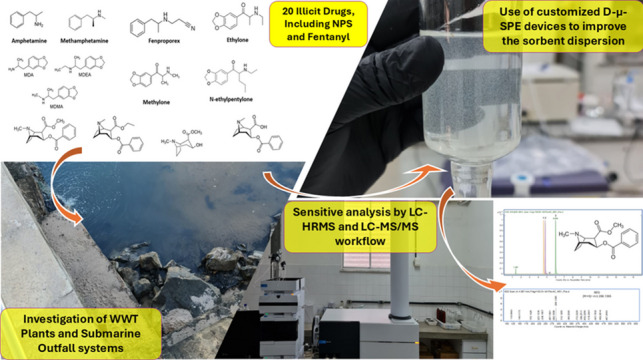

**Supplementary Information:**

The online version contains supplementary material available at 10.1007/s00216-026-06520-3.

## Introduction

Illicit drug use has been on the rise globally, particularly with a notable increase in consumption in developing countries. According to the 2023 World Drug Report, approximately 219 million people used cannabis in 2021, while 60 million used opioids, and 22 million engaged in cocaine consumption worldwide [1, 2]. Fentanyl, a powerful synthetic opioid, has been massively used in the North American markets and has caused almost 90,000 deaths [2, 3]. Owing to the elevated rates of illicit drug consumption, residues of these substances are frequently found in surface water and wastewater systems, mainly cocaine, methamphetamine, cannabis, opioids, and their metabolites [1, 2]. The improper disposal and excretion by the human body are the primary sources of discharge in wastewater systems [4, 5]. Although a significant portion of wastewater contaminants, including microbiological agents and certain chemicals, can be effectively removed by wastewater treatment plants (WWTPs) or urban submarine outfall systems (SOs), residual hazardous substances may persist in the treated wastewater [6–8]. Recent papers reported the presence of chemical substances at trace levels, such as pharmaceuticals and illicit drugs in water bodies near SOs, which are a cause for concern, since these substances are classified as emerging pollutants and may affect water quality and aquatic life [8, 9].

One of the primary challenges in wastewater analysis lies in the low concentrations of illicit drugs, which may range from nanograms per liter to a few micrograms per liter [10], alongside the high levels of dissolved organic matter and interfering compounds. Addressing these challenges may necessitate additional steps in sample preparation, such as extraction and preconcentration, besides a cleanup step to eliminate or mitigate interferences and matrix effects. Recently, techniques, such as liquid-phase microextraction [11, 12], thin-film microextraction [13], automated solid-phase extraction [14], passive sampling devices [15], modified QuERChERS [16], and direct injection [17], have been proposed to determine illicit drugs and pharmaceuticals in wastewater. However, most of the published papers [18–21] report the use of solid-phase extraction (SPE) using conventional cartridges or syringes to extract and preconcentrate these substances. Despite its simplicity, SPE has some disadvantages, such as low sample throughput, and sorbent batch-to-batch variation [22], and frequently requires an analyte concentration step after solvent elution.

Dispersive solid-phase extraction (D-SPE) has emerged as a simple and attractive alternative for extracting and preconcentrating organic compounds in complex aqueous samples. In this technique, the sorbent is dispersed into a sample solution, thereby increasing the surface area and enabling rapid sorption equilibrium [23]. Initially, it was developed exclusively for cleanup purposes, wherein an appropriate sorbent is added to aqueous samples to remove interfering compounds such as pigments, sugars, and lipids while the analytes remain in the liquid phase [23, 24]. Subsequently, the technique was employed with a focus on extracting and preconcentrating the analytes onto the sorbent. More recently, the D-µ-SPE has emerged as a miniaturized version of D-SPE to extract analytes using a small amount of sorbent (in the µg to mg range) [25]. The main advantage of D-µ-SPE is the possibility of employing different sorbents, such as carbon-based, magnetic and molecularly imprinted polymer sorbents, as well as distinct sorbent mass amounts, which may vary from 10 to 100 mg.

Recent papers have demonstrated the efficacy of D-µ-SPE in extracting illicit drugs from wastewater, predominantly employing functionalized nanosorbents [26–28]. Nevertheless, the dispersion of these sorbents typically occurs in conventional glassware such as beakers, glass tubes, or even Erlenmeyer flasks, potentially reducing sample throughput or decreasing accuracy [29, 30]. In addition, selective nanostructured sorbents obtained through synthesis may be less accessible to many laboratories [25]. Moreover, the use of D-µ-SPE coupled with HPLC systems equipped with spectrophotometric detectors or mass-spectrometric detectors equipped with single-quadrupole analyzers may not provide the necessary resolution and/or mass accuracy to adequately investigate the complex chemical dynamics of illicit drugs and their metabolites in wastewater [31, 32].

Over the past year, the number of published studies reporting the application of liquid chromatography coupled to high-resolution mass spectrometry (LC-HRMS) for untargeted and suspect screening of illicit drugs and pharmaceuticals in wastewater has increased substantially. One of the main advantages of LC-HRMS in the environmental monitoring of emerging contaminants is its ability to perform both untargeted and suspect screening of a wide range of compounds without the need for authentic standards, including previously unknown substances, while also enabling retrospective data analysis [33, 34]. This capability is particularly important in wastewater-based studies, where samples are no longer available for reanalysis. Recent studies have reported the combination of solid-phase extraction (SPE) with HRMS coupled to gas or liquid chromatography systems for targeted and suspect screening of illicit drugs and pharmaceuticals [34–39]. Although the use of dispersive micro–solid-phase extraction (D-µ-SPE) in combination with UHPLC–QqQ–MS has been reported [40], analytical procedures employing D-µ-SPE coupled with HRMS for the suspect screening of illicit drugs and pharmaceuticals remain scarce.

Accordingly, the central hypothesis of this study is that coupling D-µ-SPE with UHPLC-QTOF-MS and UHPLC-QqQ-MS enables both target and suspect screening of relevant illicit drugs, pharmaceuticals, and their metabolites, including compounds for which analytical standards are unavailable. In addition, the proposed approach also allows highly sensitive quantification of target analytes using authentic standards. Furthermore, the implementation of D-µ-SPE constitutes a greener alternative to conventional solid-phase extraction (SPE), as it substantially reduces sorbent and solvent consumption.

This study aimed to develop a novel dispersive micro-solid-phase extraction (D-µ-SPE) procedure combined with UHPLC–QTOF–MS and UHPLC–QqQ–MS for suspect screening and target analysis of illicit drugs and pharmaceutical substances, including their metabolites. Chromatographic and mass spectrometric conditions for both UHPLC–QqQ–MS and UHPLC–QTOF–MS were optimized to allow the determination of cocaine, amphetamine-type stimulants, synthetic cathinones, opioids, cannabinoids, and their metabolites within a single chromatographic run. Parameters affecting D-µ-SPE performance were optimized using a statistical experimental design approach. Following method validation, the proposed methodology was applied to perform a comprehensive investigation of illicit drugs and pharmaceuticals in real wastewater samples collected from Brazil’s largest coastal bay region.

## Material and methods

### Reagent and solutions

Isotopically labeled standards of cocaine-d_3_ (99.4%), methamphetamine-d_11_ (99.3%), norfentanyl-d_5_ (99.9%), MDMA-d_5_, and amphetamine-d_6_ (AMP-d_6_) at 1000 mg L^−1^, and the cannabinoids cannabinol (CBN, 99.1%), and cannabidiol (CBD, 99.6%), were acquired from Cerilliant (Round Rock, TX, USA). Fentanyl (FENT, 99.7%) was purchased from LGC Standards (Teddington, Middlesex, UK). A mix standard solution containing ecgonine methyl ester (EME), benzoylecgnonine (BEG), cocaine (COC), norcocaine (NOR), and cocaethylene (COET) in acetonitrile at 10 mg L^−1^ and a mix solution containing 3,4-methylenedioxyamphetamine (MDA), 3,4-methylenedioxymethamphetamine (MDMA), 3,4-methylenedioxy-methylbutanamine (MBDB), 3,4-methylenedioxy-ethylamphetamine (MDEA), fenproporex (FEN), methylone (MET), ethylone (ETH), and *N*-ethylpentylone (NEP), and clobenzorex (CBZ), amphetamine (AMP), methamphetamine (METH) in methanol at 10 mg L^−1^ were kindly donated by the Forensic Toxicology Laboratory of the Scientific Police of the State of Sergipe (Brazil).

An ultrapure water system Milli-Q Elix (Merck Millipore, Burlington, USA) equipped with a Q-POD water remote dispenser was used to obtain ultrapure water (18.2 MΩ cm and total organic carbon < 3 µg L^−1^). All organic solvents used in this study were LC-MS grade. Ultrapure methanol (MeOH) was purchased from Merck (Darmstadt, Germany). Acetonitrile (ACN) was purchased from Sigma-Aldrich (St. Louis, MO, USA). LC-MS-grade formic acid (98%) LiChropur was purchased from Merck (Darmstadt, Germany), and ammonium formate was acquired from Êxodo Científica (São Paulo, Brazil). The OASIS MCX Plus SPE cartridges (60 µm particle diameter, 200 mg) were purchased from Waters (Milford, MA, USA).

A mixed working solution was prepared by diluting the analytical standards in methanol to a concentration of 1.0 mg L^−1^. An analyte-free sample was prepared by passing 2 L of a given wastewater sample through a solid-phase extraction (SPE) system containing two connected OASIS MCX cartridges coupled to a Gilson MiniPlus 3 peristaltic pump (Madison, WI, USA) operating at a flow of 4 mL min^−1^. The resulting analyte-free solution was initially analyzed using the UHPLC-MS/MS system to verify the presence of any target analyte residues. No peaks corresponding to the target analytes were detected. Consequently, the solution was used to construct external matrix-matched calibration curves at ten concentration levels (0.5, 1.0, 5.0, 10, 25, 50, 75, 100, 200, and 400 ng L⁻^1^) using the proposed D-µ-SPE procedure. Additionally, a separate set of calibration curves was prepared with an extended linear range (10, 50, 100, 500, 1000, 5000, 10,000, and 15,000 ng L⁻^1^) to quantify analytes present at higher concentrations in wastewater samples.

### Wastewater sampling

Fourteen wastewater samples were collected from wastewater treatment plants and primary wastewater outfalls in the coastal regions of Salvador and Porto Seguro, cities located in the State of Bahia, northeastern Brazil. These cities are major tourist destinations for visitors from different regions of Brazil and from other countries, particularly because they host one of the largest street carnivals in the world. Samples were collected in 500 mL polyethylene flasks and transported to the laboratory on the same day under refrigerated conditions. Upon arrival, the samples were immediately acidified with 12 mol L⁻^1^ HCl to pH 2 using a pH meter (Mettler Toledo, Columbus, OH, USA). Subsequently, the samples were filtered through a 0.7 µm glass fiber filter (Merck, Darmstadt, Germany) using a vacuum-operated Millipore filtration system. After filtration, the samples were stored at − 20 °C until analysis. Analyses were performed within two weeks. When analysis was not feasible within this period, the samples remained frozen at − 20 °C in accordance with preservation protocols commonly reported in the literature [41, 42].

### UHPLC-QqQ-MS and UHPLC-QTOF-MS analysis conditions

The separation, identification, and quantification of the target analytes were performed using an Agilent 1260 Infinity II UHPLC system coupled to a triple quadrupole mass spectrometer Agilent Ultivo System, equipped with a quaternary pump system, an electrospray (ESI) and Agilent Jetstream (AJS) high-sensitivity ion source (Agilent Technologies, Santa Clara, CA, USA). A reversed-phase InfinityLab Poroshell 120 EC-C18 column (100 mm × 3.0 mm × 2.7 µm, Agilent Technologies, Santa Clara, CA, USA) was employed in the analyte’s separation, according to the following mobile phase gradient condition: starting with 95% solvent A (ultrapure water/0.1% formic acid) and 5% solvent B (methanol/0.5 mmol L^−1^ ammonium formate) at 0 min, increase to 85% of B at 7 min, 100% B at 15 min, holding this condition for 1 min. The column temperature was kept at 40 °C. The flow rate of the mobile phase was fixed at 0.4 mL min^−1^, and a volume of 2 µL of the sample was injected. The autosampler temperature was kept at 6 °C to avoid analyte degradation. The AJS ion source was operated in positive mode. The ionization parameters were dry gas temperature (200 °C), sheath gas temperature (400 °C), dry gas flow (8 L min^−1^), sheath gas flow (10 L min^−1^), nebulizer pressure (35 psi), capillary voltage (3 kV), and nozzle voltage (1 kV). Nitrogen was used as collision gas in the collision cell. Target acquisition was performed using two dynamic multiple reaction monitoring (dMRM) transitions for each compound, as shown in Table S1.

LC–HRMS analysis was performed using an Agilent 1290 Infinity II Binary LC system connected to an Agilent 6545B Accurate-Mass Q-TOF mass spectrometer equipped with Agilent Jet Stream technology (AJS) for electrospray ionization (Agilent Technologies, Santa Clara, CA, USA). An amount of 2 µL from each sample was loaded onto a C_18_ column (InfinityLab Poroshell 120 EC-C18 (100 mm × 3.0 mm × 2.7 μm, Agilent Technologies, Santa Clara, CA, USA)) and maintained at 40 °C. To conduct suspect screening analysis using the LC-QTOF-MS system, it was necessary to perform a slight change in the elution gradient. The UHPLC was operated at a flow rate of 400 μL min^−1^, and with the mobile phase system (solvent A: ultrapure water containing 0.1% formic acid; solvent B: 100% methanol containing 0.5 mmol L^−1^ ammonium formate) and eluent gradient (5% B for 0 min; 5–85% B in 0–7 min; 85–95% B in 7–15 min; 95% B in 16 min).

For the HRMS analysis, data was acquired using two sequential methods: the first in positive mode and the second in negative mode. The full-scan data was obtained at 100 to 600 m*/z* scanning range (3.0 spectra s^−1^), while Auto MS/MS data acquisition was carried out at 70 to 600 m/z, MS/MS scan rate at 2 spectra s^−1^. The UHPLC-QTOF-MS parameters were: a drying gas temperature of 200 °C, a drying gas flow rate of 8.0 L min^−1^, a sheath gas temperature of 400 °C, a sheath gas flow rate of 10.0 L min^−1^, nebulizer gas pressure of 35 psi, skimmer voltage of 45 V, octupole RF of 750 V, fragmentor voltage of 100 V, capillary voltage of 4.0 kV, and nozzle voltage of 1.0 kV. Ultrapure nitrogen (99.999%) was used as the collision gas. Collision energies of 10, 20, and 40 eV were applied for selective fragmentation of the analytes in Auto MS/MS mode. Agilent MassHunter Workstation software (version 10.0) was used for data acquisition, spectral processing, and qualitative analysis.

### Optimization of UHPLC-QTOF-MS and UHPLC-QqQ-MS conditions

The analytical strategy adopted for method optimization initially involved optimizing the UHPLC-QqQ-MS conditions using authentic analytical standards for the target analytes, followed by the transfer and adjustment of the optimized parameters to the UHPLC-QTOF-MS system. Notably, the same chromatographic column, mobile-phase composition, and ionization conditions were employed in both instruments to ensure methodological consistency and comparability of results.

The chromatographic separation and ionization of the target analytes by UHPLC–QqQ–MS were optimized by evaluating different mobile-phase compositions. Initially, the chromatographic conditions reported by Cunha et al.[43] were adopted and subsequently adjusted to achieve optimal analyte separation. Thereafter, the influence of mobile-phase composition on the analytical response (peak area) was investigated. The aqueous phase (A) was fixed as water containing 0.1% formic acid (v/v) throughout all experiments, whereas the organic phase (B) was evaluated under the following conditions: methanol containing 0.5 mmol L⁻^1^ ammonium formate; acetonitrile containing 0.5 mmol L⁻^1^ ammonium formate; methanol containing 0.1% formic acid; acetonitrile containing 0.1% formic acid; methanol containing 0.1% formic acid and 0.5 mmol L⁻^1^ ammonium formate; and acetonitrile containing 0.1% formic acid and 0.5 mmol L⁻^1^ ammonium formate. For all experiments, a mixed solution containing all target analytes at a concentration of 500 ng mL⁻^1^ was injected six times (*n* = 6) for each mobile-phase composition.

After establishing the optimal mobile-phase composition, the ionization-source parameters were further optimized. Two different electrospray ionization sources were compared: the Agilent conventional ESI source (PN# G1948B) and the Agilent Jet Stream technology source (AJS, PN# G1958-67638). The AJS source uses superheated nitrogen to enhance droplet desolvation and ion generation while simultaneously reducing spray expansion. This effect improves ion transfer into the desolvation line, thereby increasing the analytical response.

### D-µ-SPE extraction optimization

The analytical procedure developed in this study involved the use of a custom-designed glass extraction device combined with an innovative glass desorption apparatus specifically designed to complement it. This desorption device enabled sequential sorbent filtration and desorption within the same system, thereby reducing sorbent losses while simultaneously improving sample throughput.

After confirming the suitability of the D-µ-SPE device for the extraction procedure, the next step focused on investigating the factors affecting dispersive solid-phase extraction efficiency. In the initial stage, a resolution III fractional factorial design was employed to screen the significant variables. These factors were divided into two groups: (i) extraction factors and (ii) desorption factors.

The extraction factors included extraction time (10–40 min), sorbent type (OASIS MCX or OASIS HLB), sorbent amount (50–150 mg), and NaCl percentage (salting-out effect). The desorption factors comprised desorption time (1–5 min), desorption solvent volume (methanol, 1–5 mL), and the percentage of ammonium hydroxide in methanol (5–10%, v/v). A total of eleven experiments were performed in random order, including three replicates at the central point. In all extraction trials, 100 mL of sample solution containing all analytes at 100 ng L⁻^1^ each was used.

The significant factors identified during the screening stage were selected for further optimization using response surface methodology (RSM). The Doehlert design was chosen because of its recognized efficiency [44].

### QA/QC and method validation

Precautionary measures were taken prior to analysis to minimize the risk of cross-contamination. Blank analyses were performed, including instrumental, reagent, and method blanks. Instrumental blanks were assessed by injecting the LC-MS/MS mobile phase. Reagent blanks were evaluated by analyzing the organic solvents used throughout the procedure, whereas method blanks were assessed by extracting ultrapure water according to the prescribed procedure in the absence of analytes [45]. None of these blank tests showed detectable levels of the target analytes.

Isotopically labeled standards such as amphetamine-d_6_, MDMA-d_5_, norfentanyl-d_5_, methamphetamine-d_11_, and cocaine-d_3_ were used as surrogate standards. They were added to the samples and blanks before the extraction at a final concentration of 200 ng L^−1^. The recoveries of the surrogate standards added to the samples were evaluated in each batch. The recoveries ranged from 78.2 to 97.4% for all target analytes.

The analytical procedure was subjected to validation according to the analytical parameters recommended by IUPAC for “in-house” validation [46, 47]. The matrix effect (ME), linear range and linearity, limit of detection (LOD), limit of quantification (LOQ), precision, and trueness (recovery assay) were evaluated. In addition, the enrichment factor (EF) and extraction recovery (ER) were investigated to assess the extraction efficiency. Details for method validation and calculations can be found in Supplementary Information.

### Wastewater sample analysis

Samples were analyzed under the optimized and validated method conditions. For extraction, 100 mL of wastewater was spiked with 100 µL of a surrogate standard mixture to yield a final concentration of 100 ng L⁻^1^. The sample was then transferred to a glass extraction device containing 50 mg of OASIS MCX sorbent, previously weighed on an analytical balance (± 0.1 mg). The device was sealed with a glass lid and subjected to vortex-assisted extraction for 30 min at 1000 rpm.

Subsequently, the desorption device was connected to the extraction unit, and the assembled system was coupled to a vacuum filtration setup. The sample solution was filtered, allowing the sorbent to be retained at the bottom of the desorption device. For analyte desorption, 1.25 mL of 5% ammonium hydroxide in methanol was added to the device containing the sorbent, followed by vortex agitation for 3 min at 2500 rpm.

**Fig. 1:**
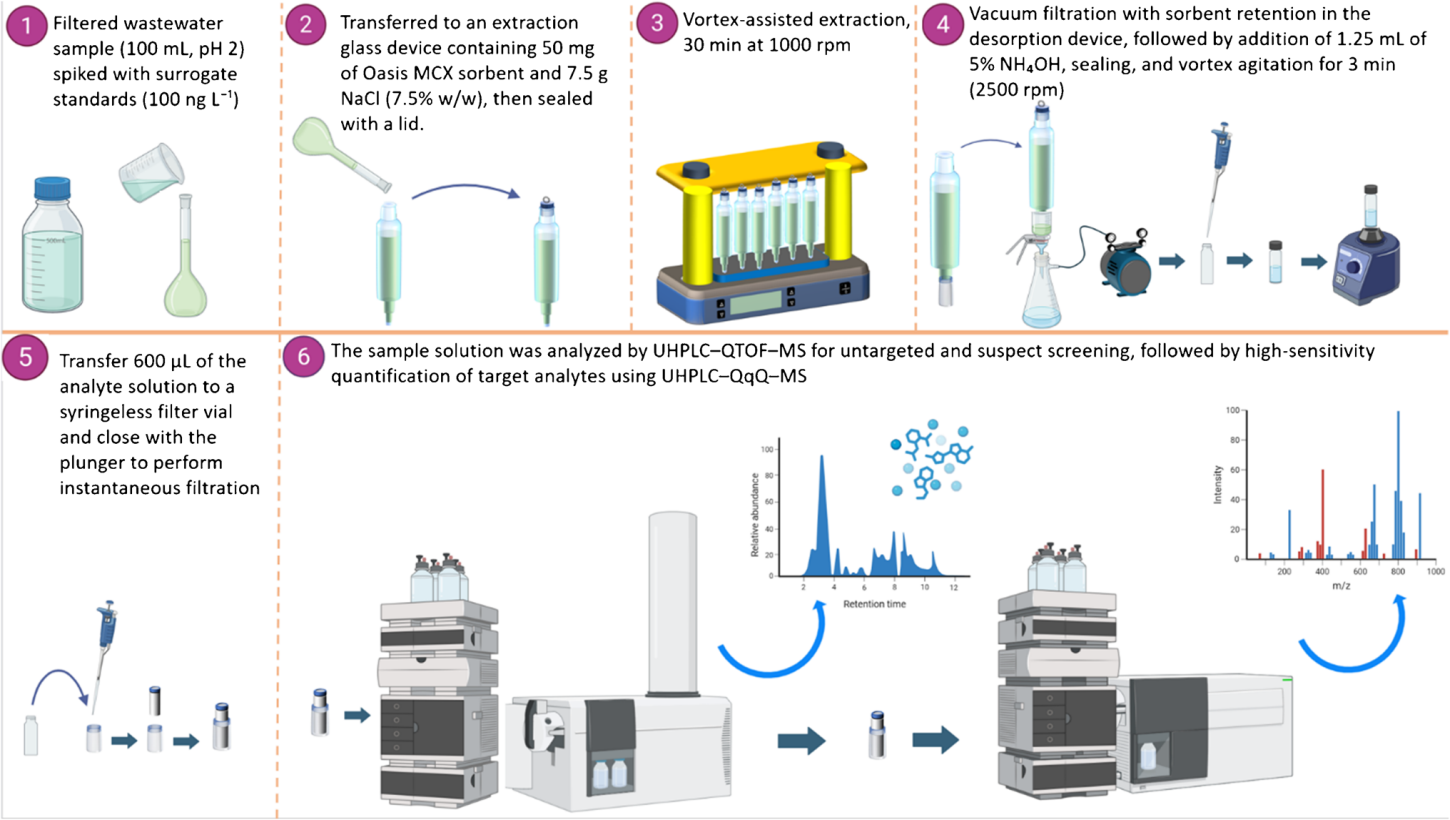
Illustrative scheme of the developed D-µ-SPE workflow

After desorption, approximately 600 µL of the extract was transferred to a Whatman MiniUniprep syringeless filter unit (Cytiva, USA). The solution was immediately filtered and placed in the autosampler of the LC–HRMS system for injection and suspect screening analysis. A subsequent injection into the LC–MS/MS system was performed for quantification of the target compounds. An illustrative scheme of the overall workflow is presented in Fig. 1.

### Suspect screening workflow

Suspect screening was performed using Agilent MassHunter Qualitative Analysis Software version 10.0. Data were processed without noise filtering or additional spectral pre-treatment. Both positive and negative ionization modes were evaluated; for the positive mode, adducts commonly formed with the mobile phase or sample matrix were considered, including [M+H]⁺, [M+Na]⁺, and [M+NH_4_]⁺. Molecular formula generation included elements typically present in illicit drugs and pharmaceuticals (C, H, O, N, S, and Cl). Quality assurance was based on the use of procedural blanks consisting of ultrapure water subjected to the same extraction protocol as the samples. Features detected in procedural blanks were considered background contaminants and removed from the suspect list prior to further evaluation.

Database searches were conducted using accurate-mass tolerances of 10 ppm for precursor ions and 15 ppm for fragment ions. A retention-time window of ± 0.25 min was applied when comparing experimental features with database entries. Acquired spectra were matched against the Agilent PCDL Spectral Library, and suspect identification was performed using the Compound Discovery and Compound Mining algorithms from Auto MS/MS data.

Data-dependent acquisition was employed, in which the five most intense precursor ions detected in the MS survey scan (m/z 70–600, 3 spectra s⁻^1^) were automatically selected for MS/MS fragmentation. Product-ion spectra were acquired over m/z 70–600 at 2 spectra s⁻^1^, using an isolation width of 4 amu. Variable collision energies of 10, 20, and 40 eV were applied for selective fragmentation of the analytes in Auto MS/MS mode. A TIC intensity threshold of 1000 counts was applied for MS/MS triggering in both ionization modes. Fragment ions were only considered when presenting a signal-to-noise ratio greater than 5. Confirmation of suspect compounds required a minimum match confidence of 70%. Identification confidence levels followed the Schymanski et al. [48] classification. Features with accurate mass within tolerance were assigned to level 5. Candidates additionally exhibiting agreement with the theoretical isotopic pattern were classified as level 4. Compounds supported by exact mass, isotopic pattern, and experimental MS/MS spectra were assigned to level 3. Matches based solely on compound mass lists were considered level 2b, whereas those supported by spectral library matching were classified as level 2a. Compounds confirmed using authentic analytical standards were reported as level 1.

### Data analysis

Microsoft Excel (Office 365, Redmond, WA, USA) was used for mathematical calculations. Statistica 7.0 (Tulsa, OK, USA) was employed to evaluate the goodness-of-fit of the mathematical models obtained from the experimental design using analysis of variance (ANOVA, *p* < 0.05) and to construct response surface plots. Model significance and lack of fit were assessed by F-tests, calculated as the ratios MS_R_/MS_r_ (mean square of regression over mean square of residuals) and MS_lof_/MS_pe_ (mean square of lack of fit over mean square of pure error), respectively, and compared with the tabulated F value at a 5% significance level. OriginPro 2026 (OriginLab, Northampton, MA, USA) was used for graphical representation.

## Results and discussion

### Optimization of the chromatography and mass-spectrometry conditions

Details concerning the optimization of the mobile phase composition are provided in the Supplementary Information (Figs. S1–S4). In addition, the type of ionization source was considered a critical parameter for achieving high sensitivity for the target analytes. Therefore, the influence of the ionization source on the analytical response was systematically evaluated. The results demonstrated that the use of the AJS ionization source increased method sensitivity by up to one order of magnitude for all analytes (Fig. S5). Based on these findings, the AJS source was selected for subsequent analyses using both the UHPLC-QTOF-MS and UHPLC-QqQ-MS systems. The retention times and precursor ions of the target illicit drugs are presented in Table S2.

### Optimization of the D-µ-SPE extraction conditions

**Fig. 2:**
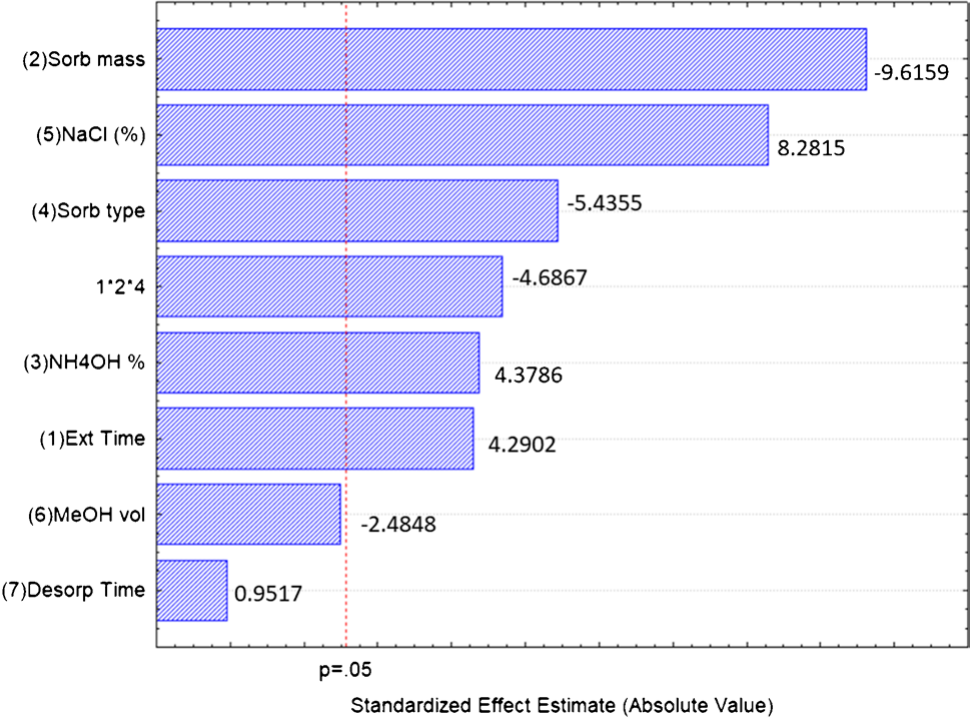
Pareto chart for standardized effects at 95% confidence level for the resolution III fractional factorial design

The influencing factors on D-µ-SPE extraction were thoroughly investigated utilizing a resolution III fractional factorial design [49]. The ANOVA (*p* < 0.05) was applied to evaluate the fit of the linear model. The results indicate that most of the variance in the experimental response was explained by the mathematical model and not by the residues (R^2^ = 0.9809). There is no evidence of a lack of fit at a 95% confidence level, and the residuals were low and randomly distributed. The analysis of the Pareto graph of standardized estimated effects (Fig. 2) revealed that the factors “sorbent mass,” “NaCl concentration,” “sorbent type,” “NH_4_OH concentration,” and “the extraction time” were statistically significant at a 95% confidence level.

Notably, the factor “sorbent mass” demonstrated a pronounced negative effect, suggesting that using lower amounts of sorbent led to an increase in the peak areas of the analytes. Using a greater sorbent mass requires a substantial increase in the volume of desorption solvent or an increase in solution pH. On the other hand, the factor “percent of NaCl” exhibited a strong positive effect on the analytical response, indicating an increase in the analyte peak areas when the NaCl percentage was increased. This can be explained by considering the salting out effect, which occurs when employing elevated salt concentrations, such as NaCl. The solubility of polar organic substances in the aqueous medium can be decreased in the presence of sodium and chloride ions [50]. Consequently, the partitioning equilibrium is altered to favor the interaction between the analytes and the sorbent.

The qualitative factor “sorbent type” exhibited a negative effect, indicating that the sorbent studied in the lowest level of the experimental design was more effective in extracting the analytes. In other words, the mixed-mode polymeric sorbent OASIS MCX containing the alkylbenzenesulfonate group as a cation exchange agent provides better results than the OASIS HLB (hydrophilic-lipophilic balanced sorbent). This can be explained by considering the basic properties of most target analytes. The basic drugs are protonated in an acidic medium (pH 2), acquiring a positive charge. Thus, the interaction with the alkylbenzenesulfonate cation-exchange group is highly favored, increasing analyte sorption. On the other hand, the factor “extraction time” demonstrated a positive effect, indicating that a longer extraction time is required for the analytes to attain the sorption equilibrium.

Regarding the desorption factors, such as “desorption time,” “volume of MeOH,” and “percentage of NH_4_OH,” it was observed that only “percent of NH_4_OH” was significant, exhibiting a positive effect on the response. In other words, adding NH_4_OH to methanol above 5% improves the desorption step, leading to an increase in the analytes’ peak areas. However, the pH of the methanolic solution is also increased. This may be challenging when using C_18_ chromatographic columns with a restricted pH range (pH 2–8). For these reasons, we decided to fix the NH_4_OH concentration at 5%, as it is routinely used in most published papers. The non-significant factors “desorption time” and “volume of methanol” were fixed at the center-point values of the experimental design.

The factors "OASIS MCX mass," "percentage of NaCl," and "extraction time," which influenced the analytical response, were investigated using response surface methodology (RSM) to determine optimal conditions. A Doehlert matrix consisting of three variables was employed (Table S3A). ANOVA (*p* < 0.05) was conducted to evaluate the adequacy of the quadratic model fit (Table S3B). The ANOVA results revealed that the MSR/MSr ratio (calculated *F* = 1.39) was below the critical *F*-value (4.10), indicating that the quadratic model was not statistically significant at the 95% confidence level. This may be attributed to the low variability in the responses when different levels were tested. However, no evidence of lack of fit was observed, as the MS_lof_/MS_pr_ ratio (1.11) was lower than the critical F value (9.28). The obtained quadratic model could not be used for optimum point prediction in this condition. Consequently, a visual inspection of the multiple responses was performed in the Doehlert design data matrix (Table S3A), where a maximum response was observed in the center point of the experimental design (50 mg OASIS MCX, 7.5% NaCl % wt., and 30 min of extraction time). Under these conditions, we performed extraction tests in triplicate, and extraction recoveries ranging from 60 to 109% were obtained for most of the target analytes. Thus, we decided to keep these conditions for further steps.

### Validation of the analytical procedure

**Fig. 3:**
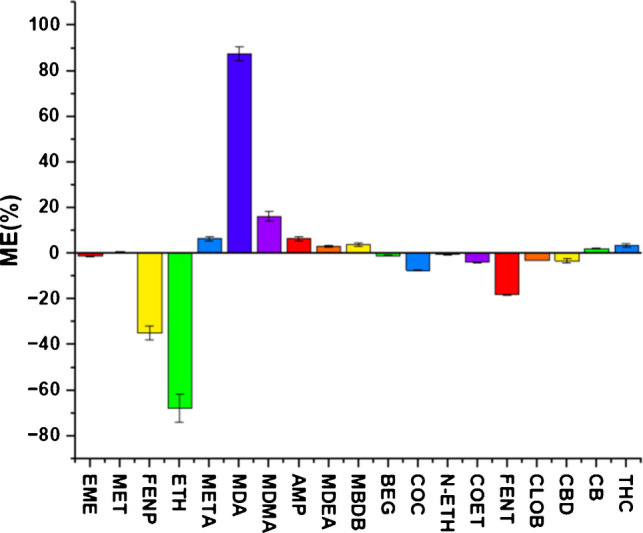
Matrix-effect study of D-µ-SPE by UHPLC-QqQ-MS at 50 ng mL^−1^. The ME was expressed as mean (n = 4) and standard deviation

The analytical parameters of the procedure developed are presented in Table 1. Concerning selectivity, no interfering compounds were observed to elute at the same retention time as the target analytes in any dMRM transitions. The matrix effect study (ME) indicated both ion suppression and signal enhancement induced by the matrix (Fig. 3).

**Table 1:** Validation parameters of the developed analytical procedure

	**Linearity**						**Precision**		**Extraction efficiency**					
**Compounds**	**Linear range** **(ng L**^**−1**^**)**	**R**^**2**^	***F*-calc** **(*F-*critical)**^**a**^	***T*-cal** **(*t-*critical)**^**a**^	**LOD** **(ng L**^**−1**^**)**	**LOQ** **(ng L**^**−1**^**)**	**Rep. (%) (n = 10)**	**Inter (%)** **(n = 30)**	**EF (EF**_**max**_** = 80)**			**ER (%)**		
AMP	5.38–400	0.9977	213,067 (2.92)	104 (2.05)	1.62	5.38	1.18	7.24	75.8	** ± **	0.2	94.7	** ± **	0.2
BEG	7.88–400	0.9913	147,060 (2.92)	54 (2.05)	2.37	7.88	4.57	5.91	73.1	** ± **	1.0	91.4	** ± **	1.0
CBN	16.7–400	0.9964	23,241 (3.89)	60 (2.059)	5.01	16.7	0.870	7.41	20.3	** ± **	1.9	25.0	** ± **	1.9
CBD	28.2–600	0.9903	34,516 (4.38)	73 (2.086)	8.46	28.2	0.830	7.43	32.9	** ± **	1.8	41.1	** ± **	1.8
CBZ	8.72–500	0.9981	990,416 (2.89)	121 (2.045)	2.62	8.72	0.920	5.32	82.3	** ± **	0.9	100	** ± **	1
COC	5.65–400	0.9964	139,313 (2.92)	83 (2.05)	1.70	5.65	1.16	2.98	75.8	** ± **	0.2	94.7	** ± **	0.2
COET	7.00–400	0.9968	199,924 (2.89)	93 (2.045)	2.10	7.00	1.44	3.53	81.1	** ± **	1.8	100	** ± **	2
EME	20.5–500	0.9975	8894 (3.10)	74 (2.145)	6.15	20.5	6.45	19.2	20.9	** ± **	2.8	26.2	** ± **	2.8
ETH	5.63–500	0.9983	477,813 (2.94)	116 (2.064)	1.69	5.63	1.40	5.73	79.8	** ± **	0.5	99.8	** ± **	0.5
FEN	4.67–400	0.9979	282,246 (2.92)	108 (2.056)	1.40	4.67	2.08	4.73	86.2	** ± **	1.4	100	** ± **	1
FENT	6.17–400	0.9971	81,189 (2.94)	93 (2.056)	1.85	6.17	1.18	4.80	82.7	** ± **	0.5	100	** ± **	0
MDA	7.87–600	0.9997	263,602 (5.59)	277 (2.064)	2.36	7.87	2.50	4.10	48.0	** ± **	2.6	60.0	** ± **	2.6
MBDB	2.00–500	0.9977	431,136 (2.92)	33 (2.056)	2.00	6.65	1.53	6.02	74.4	** ± **	1.2	93.0	** ± **	1.2
MDEA	1.51–400	0.9980	723,094 (2.92)	112 (2.056)	1.51	5.03	1.20	5.68	78.1	** ± **	0.7	97.6	** ± **	0.7
MDMA	2.73–400	0.9959	723,094 (2.92)	68 (2.101)	2.73	9.10	7.75	9.45	58.8	** ± **	2.7	73.5	** ± **	2.7
MET	1.78–400	0.9983	164,577 (2.92)	121 (2.056)	1.78	5.94	0.950	4.66	74.6	** ± **	0.1	93.3	** ± **	0.1
METH	1.55–500	0.9982	303,624 (2.94)	116 (2.064)	1.55	5.16	0.570	5.27	70.3	** ± **	0.2	87.9	** ± **	0.2
NEP	6.84–500	0.9985	192,170 (2.89)	128 (2.045)	2.05	6.84	0.730	5.55	78.6	** ± **	0.3	98.3	** ± **	0.3
NOR	5.36–400	0.9939	236,618 (2.95)	113 (2.069)	1.61	5.36	3.21	7.04	80.9	** ± **	1.2	100	** ± **	1
THC	8.74–600	0.9963	75,105 (3.63)	69 (2.101)	8.79	29.3	1.57	8.44	21.7	** ± **	0.5	27.0	** ± **	0.5

^a^5% of significance in t-Student’s distribution. *Rep.*, repeatability; *Inter*, intermediate precision; *EF*, enrichment factor; *ER*, extraction recovery

Overall, the ME values ranged from −34.8 to 87.5%. Signal suppression was observed for FENP, ETH, and FENT whereas significant signal enhancement was observed for MDA and MDMA. The remaining analytes showed moderate or insignificant ME. When employing LC-MS techniques, signal suppression represents a prevalent matrix effect. This phenomenon primarily arises due to the competition for ionization between the target analytes and either co-eluting or otherwise undetected components within the matrix at the electrospray ionization source [51, 52]. An elevated concentration of matrix components in the sample extract can detrimentally impact the ionization efficiency of the target analytes, particularly when these matrix components are present in excess [52]. Due to the issues mentioned above, matrix-matched analytical curves were employed to correct the matrix effect.

With respect to linearity, an initial assessment revealed heteroscedasticity across all examined analytical curves. This condition was characterized by a proportional increase in the standard deviation of the analytical response (*y*) as the concentration (*x*) increased [42]. Consequently, it became imperative to employ weighted least squares regression utilizing the inverse of concentration (1/*x*) to address this issue[53]. After this adjustment, the coefficient of determination (R^2^) was observed to vary from 0.9903 (CBD) to 0.9985 (NEP). To certify the appropriateness of R^2^, a *t*-test was employed for all analytical curves [45]. The results indicated that the calculated t value was much higher than the critical t value at 95% confidence (Table 1). Additionally, the application of ANOVA (*p* < 0.05) to all analytical curves demonstrated that the linear model was significant with respect to the residuals, as evidenced by the calculated *F*-value (goodness of fit), which exceeded the critical *F* value by more than 100-fold at a 95% confidence level [45]. Thus, the analytical curves were considered suitable for analyte quantification.

LOD and LOQ values ranged from 1.40 ng L⁻^1^ (FEN) to 8.79 ng L⁻^1^ (THC) and from 4.67 ng L⁻^1^ to 29.3 ng L⁻^1^ for the same compounds, respectively. In comparison, the LOQs achieved in the present study were lower than those reported by Psichoudaki et al. (2023) for AMP (6.40 ng L⁻^1^), METH (5.5 ng L⁻^1^), and BEG (15.5 ng L⁻^1^), with the exception of MDMA (3.4 ng L⁻^1^) [54]. Notably, although the authors employed the same sample volume (100 mL), sorbent material (OASIS MCX), and instrumental configuration, their methodology differed in that conventional SPE was employed for analyte extraction from the samples.

Regarding precision, RSD values for repeatability ranged from 0.83% (CBD) to 6.45% (EME), demonstrating that all target compounds exhibited RSD values below 10%. Similarly, RSD values for intermediate precision varied between 2.98% (COC) and 19.2% (EME). Considering that RSD values of up to 20% are generally considered acceptable at the concentration levels evaluated in this study, the proposed D-µ-SPE procedure can be regarded as precise [55].

With respect to the extraction performance of the proposed D-µ-SPE procedure, enrichment factors (EF) ranged from 20.3 (CBN) to 86.2 (FENT), while extraction recoveries (ER) varied from 73.5% (MDMA) to 100% (FEN, FENT, NOR, CBZ, and COET) for most of the target analytes (Table 1). However, lower ER values (≤ 60%) were observed for MDA, EME, and cannabinoids (CBN, CBD, and THC). For MDA and EME, the reduced ER can be attributed to strong interactions between these polar compounds and the cation-exchange functional groups of the OASIS MCX sorbent, which may hinder complete elution of the analytes. In the case of cannabinoids, the presence of a long alkyl chain increases molecular hydrophobicity, thereby limiting interactions with the polar functionalities and ionic-exchange sites of the sorbent.

As no certified reference material is commercially available for illicit drugs in wastewater matrices, trueness was assessed through relative recovery experiments. As shown in Table S4, relative recoveries ranged from 79.3% (FEN) to 118% (NOR) in spiked wastewater samples and from 60.7% (BEG) to 105% (NOR) in river water samples.

Overall, satisfactory relative recoveries were obtained at all evaluated concentration levels, with the exception of BEG, which exhibited a relative recovery of 60.7% at the lowest spiking level in river water. Nevertheless, BEG showed satisfactory recovery at higher concentration levels in both wastewater and river water. Although recovery values within the range of 80–120% are commonly regarded as acceptable in the literature, this criterion may be broadened to 60–120%, depending on analyte concentration and matrix complexity [55]. Accordingly, the proposed procedure can be considered accurate for the analysis of wastewater and river water samples.

### Analysis of real wastewater samples

#### Suspect screening

The initial step comprised suspect screening of potential illicit drugs and pharmaceuticals in wastewater samples using UHPLC–Q-TOF-MS. Data processing was performed with the Compound Discovery workflow, applying an MS/MS-based compound mining algorithm. The Water Screening and Veterinary Drugs libraries available in the MassHunter PCDL package were used to tentatively match experimental mass spectra with database entries.

On average, approximately 600 candidate features were detected per sample prior to data curation. Following systematic filtering and evaluation of the candidates, 35 compounds were retained and assigned identification confidence levels according to established criteria. Of these, 28.6% were confirmed at level 1 by comparison with authentic analytical standards. A further 34.3% were assigned to level 2 (2a or 2b), indicating high-confidence identification based on diagnostic MS/MS evidence and/or library matching. The remaining compounds were classified as level 3 (14.3%) or level 4 (22.9%), corresponding to tentative candidates and unequivocal molecular formula assignments, respectively. It should be emphasized that other common organic contaminants, including pesticides, personal care products, and plasticizers, were also detected. However, these substances were not included in the discussion, as the analytical focus was restricted to illicit drugs and pharmaceutical compounds. The list of compounds retained after suspect screening is summarized in Table 2.

**Table 2:** Illicit drugs and pharmaceuticals identified by UHPLC-QTOF-MS (ESI+) in real wastewater samples

Compound	Rt (min)	Molecular Formula	Precursor Theoretical [M+H]^+^ (m/z)	Measured [M+H]^+^ (m/z)	Mass error (ppm)	MS^2^ Characteristic fragment (m/z)	ΔRt (min)	Confidence level	Classification
2,6-Xylidine	2.47	C_8_H_11_N	122.0964	122.0965	0.82	79.0542	NA	4	Chemical/lidocaine metabolite
Alprenolol	3.69	C_15_H_23_NO_2_	250.1802	250.1810	3.20	91.0542	NA	2b	Non-selective beta-blocker
Ampyrone*	4.68	C_11_H_13_N_3_O	226.0951	226.0957	2.65	-	NA	4	Aminopyrine metabolite, analgesic
Amphetamine	2.78	C_9_H_13_N	136.1121	136.1120	−0.73	91.0541	0.20	1	CNS stimulant
Benzoyilecgonine	4.57	C_16_H_19_NO_4_	290.1387	290.1395	2.76	168.1019	0.03	1	Cocaine Metabolite
17-β-Estradiol (E2)	9.24	C_18_H_24_O_2_	273.1849	273.1851	0.73	255.1747	NA	3	Natural steroidal estrogen hormone
Bisoprolol	8.54	C_18_H_31_NO_4_	326.2326	326.2322	−1.23	162.0986	NA	2b	β1-selective blocker
Caffeine	4.24	C_8_H_10_N_4_O_2_	195.0877	195.0873	−2.05	138.0656	NA	2a	CNS stimulant, wastewater marker
Carbamazepine	6.81	C_15_H_12_N_2_O	237.1022	237.1031	3.80	194.0967	NA	2b	Dibenzazepine anticonvulsant
Ciprofloxacin	4.27	C_17_H_18_FN_3_O_3_	332.1405	332.1421	4.82	288.1517	NA	2b	Broad-spectrum antibiotic
Cocaethylene	5.20	C_18_H_23_NO_4_	318.1700	318.1699	−0.31	196.1326	0.25	1	Cocaine Metabolite
Cocaine	4.81	C_17_H_21_NO_4_	304.1543	304.1543	0.00	182.1176	0.12	1	Stimulant
Coumarin	5.63	C_9_H_6_O_2_	147.0441	147.0442	0.68	91.0542	NA	2a	Fragrance/flavor compound
Ecgonine Methyl Ester	5.97	C_10_H_17_NO_3_	200.1281	200.1284	1.50	91.0540	0.07	1	Cocaine Metabolite
Embutramide	8.48	C_17_H_27_NO_3_	294.2064	294.2073	3.06	221.1168	NA	3	Sedative for veterinary euthanasia
Etilefrine	1.67	C_10_H_15_NO_2_	182.1176	182.1173	−1.65	81.0691	NA	3	Adrenergic agonist for hypotension
Fentanyl	5.63	C_22_H_28_N_2_O	337.2275	337.2270	−1.48	105.0321	0.14	1	Synthetic opioid analgesic
Ibesartan	7.54	C_25_H_28_N_6_O	429.2397	429.2412	3.49	207.0917	NA	2a	ARB antihypertensive
Lindocaine	7.67	C_14_H_22_N_2_O	235.1805	235.1804	−0.43	-	NA	4	Local anesthetic, antiarrhythmic
Losartan	7.24	C_22_H_23_ClN_6_O	423.1695	423.1691	−0.95	157.0978	NA	2a	ARB antihypertensive
MBDB	4.97	C_12_H_17_NO_2_	208.1332	208.1333	0.48	135.0782	0.2	1	Psychoactive/Recreational Drugs
MDMA	4.01	C_11_H_15_NO_2_	194.1176	194.1177	0.52	135.0439	0.14	1	Psychoactive stimulant
Mephenesin	6.00	C_10_H_14_O_3_	183.1016	183.1010	−3.28	-	NA	4	Muscle Relaxant
Methylphenidate (Ritalin)	4.49	C_14_H_19_NO_2_	234.1489	234.1484	−2.14	-	NA	4	CNS stimulant for ADHD
Minoxidil	4.26	C9H15N5O	210.1349	210.1342	−3.33	150.0545	NA	2b	Vasodilator, treatment of hair loss
Nicotine	1.79	C_10_H_14_N_2_	163.1230	163.1234	2.45	117.0570	NA	2a	CNS stimulant
Norcocaine	5.02	C_16_H_19_NO_4_	290.1387	290.1376	−3.79	168.1014	0.03	1	active metabolite of cocaine
Phenazone	4.77	C_11_H_12_N_2_O	189.1022	189.1019	−1.59	104.0496	NA	2a	Anti-inflammatory/Analgesic
Atenolol	8.85	C_14_H_22_N_2_O_3_	267.1703	267.1715	4.49	190.0862	NA	3	β-blocker
Propiram	7.70	C_16_H_25_N_3_O	276.2070	276.2076	2.17	-	NA	4	Opioid analgesic
Tapentadol	6.50	C_14_H_23_NO	222.1852	222.1858	2.70	93.0691	NA	2b	Opioid analgesic
THC	11.49	C_21_H_30_O_2_	315.2319	315.2325	1.90	81.0696	0.05	1	Psychoactive/Recreational Drugs
11-Hydroxy-delta9-THC	10.58	C_21_H_30_O_3_	331.2268	331.2265	10.57	123.1180	NA	3	THC metabolite
Tramadol	7.28	C_16_H_25_NO_2_	264.1958	264.1965	2.65	-	NA	4	Atypical opioid analgesic
Zopiclone	9.18	C_17_H_17_ClN_6_O_3_	389.1123	389.1121	−0.51	-	NA	4	Sedative-hypnotic

*ΔRt (min)*, retention time variation regarding the analytical standard. *Precursor detected as [M+Na]^+^

**Fig. 4:**
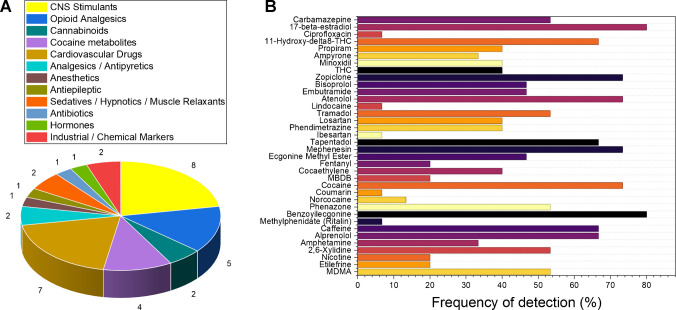
Compounds detected by the suspect screening approach in real wastewater samples. **A** Distribution of the identified compounds according to their pharmacological categories. **B** Frequency of detection of illicit drugs and pharmaceutical compounds across the 15 analyzed wastewater samples

Figure 4A presents the distribution of the detected compounds according to their pharmacological classification and Fig. 4B shows the frequency of detection of the detected compounds in the analyzed wastewater samples. The majority of the tentatively identified substances were assigned to the categories of central nervous system (CNS) stimulants and cardiovascular drugs. The CNS stimulant group comprised illicit drugs, including amphetamine, MDMA, MBDB, and cocaine, as well as licit stimulants such as caffeine and nicotine, which are widely consumed and commonly introduced into the sewage system through domestic wastewater. In addition, prescription stimulants were detected, including methylphenidate (commercially available as Ritalin) and phendimetrazine. Methylphenidate is extensively prescribed for the treatment of attention deficit hyperactivity disorder (ADHD), whereas phendimetrazine is classified as an anorectic agent with central stimulant activity. Together, these findings highlight the coexistence of illicit drugs, lifestyle-related stimulants, and therapeutic agents within the investigated wastewater samples.

Cardiovascular agents comprised β-blockers (alprenolol, atenolol, and bisoprolol), angiotensin II receptor blockers used as antihypertensives (losartan and irbesartan), and the vasodilator etilefrine. The detection of these pharmaceuticals in wastewater likely reflects their widespread therapeutic use, consistent with the high prevalence of cardiovascular diseases, which remain the leading cause of premature mortality in Brazil [56]. Particular attention should be given to the occurrence of opioid analgesics, including fentanyl, tapentadol, and tramadol. These compounds are potent centrally acting opioids, generally reserved for the management of moderate to severe pain, often in hospital settings or specialized care. Their presence in wastewater is therefore of concern, as it may indicate substantial consumption and potential misuse. Notably, a recent study describing elevated tramadol concentrations in urban wastewater suggested possible non-medical use, supporting its classification as an emerging substance of abuse in certain contexts [18]. Other common detected contaminants include cocaine metabolites, sedatives, chemical markers, antiepileptics, hormones, and antibiotics. These finds are consistent with other recent studies which employed SPME combined with HRMS for investigation of emerging contaminants in aquatic environments [18, 35].

**Fig. 5:**
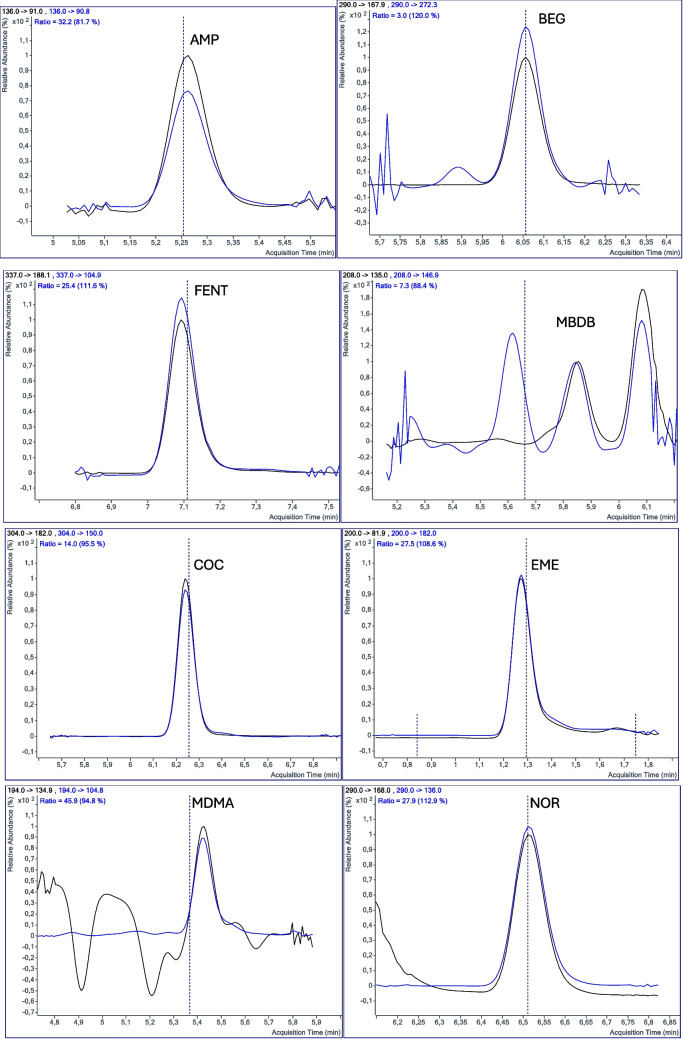
Retention times and multiple reaction monitoring (MRM) transitions of the major illicit drugs and fentanyl quantified in a wastewater sample collected in Porto Seguro are shown. The compounds are listed in alphabetical order: AMP (5.25 min), BEG (6.06 min), COC (6.26 min), EME (1.26 min), FEN (7.10 min), MBDB (5.85 min), MDMA (5.47 min), and NOR (6.51 min)

With respect to the detection frequency (Fig. 5B), 17β-estradiol and benzoylecgonine (BEG) were identified in 80% of the wastewater samples. 17β-Estradiol is the primary biologically active estrogen in women of reproductive age, whereas BEG represents the main urinary metabolite of cocaine [41, 57]. Given that human excretion via domestic wastewater constitutes the principal input pathway for these compounds into sewer systems, their high detection frequency in the investigated samples was expected. On the other hand, COC, mephenesin, EME, atenolol, and zopiclone were detected in 70% of the samples. In addition, alprenolol, caffeine, tapentadol, tramadol, 11-hydroxy-Δ9-tetrahydrocannabinol, and carbamazepine were present in more than 60% of the samples analyzed. The concurrent detection of 11-hydroxy-Δ9-tetrahydrocannabinol and THC (Δ9-tetrahydrocannabinol) may indicate cannabis consumption within the contributing population, as both compounds are associated with its metabolic excretion profile [58].

#### Quantification of illicit drugs in real wastewater samples

In the second step, the illicit drugs and pharmaceuticals confirmed at level 1 by HRMS for which authentic analytical standards were available, were quantified in the wastewater samples using the validated UHPLC-QqQ-MS method. Out of the twenty target analytes, eleven (COC, BEG, NOR, EME, FEN, MBDB, MDMA, AMP, CBN, CBD, and THC) were quantified in the wastewater samples. Cocaine and its metabolites were found to be the primary compounds detected in the samples, as previously indicated by the UHPLC-QTOF-MS suspect screening analysis. The concentration of COC ranged from 9.56 ng L^−1^ to 2392 ng L^−1^, whereas its metabolites BEG, NOR, COET, and EME exhibited concentrations ranging from 13.7 ng L^−1^ to 11,140 ng L^−1^, from 6.30 ng L^−1^ to 6825 ng L^−1^, from 12.7 ng L^−1^ to 81.6 ng L^−1^, and from 12.2 ng L^−1^ to 327 ng L^−1^, respectively. A multiple reaction monitoring (MRM) chromatogram of the quantified illicit drugs and pharmaceuticals is presented in Fig. 5.

COC is the second most consumed drug in Brazil and one of the most consumed worldwide; thus, high levels of this drug in wastewater were expected [2, 59]. For comparison, the concentrations of COC and BEG found in this work were higher than those determined by Roveri et al. [8], who investigated the occurrence of COC in wastewater samples from Guarujá City, São Paulo, Brazil. They found COC and BEG concentrations ranging from 0.3 ng L^−1^ to 0.6 ng L^−1^ and from 0.3 ng L^−1^ to 1.7 ng L^−1^. The metabolites BEG and EME are the main products of COC metabolism and their presence in the wastewater samples indicates the excretion of COC [41, 60]. The presence of the metabolite COET indicates the ingestion of cocaine (COC) in conjunction with alcoholic beverages, as the metabolic pathway of cocaine is altered in the presence of ethanol. Rather than undergoing hydrolysis with water, cocaine undergoes interesterification with ethanol to produce COET [60, 61].

The assessment of COC/BEG ratios has been used to determine the primary source of COC input in sewage systems. BEG is a byproduct of COC hydrolysis, and its high concentration (resulting in a low COC/BEG ratio) in wastewater can indicate excreted COC rather than unmetabolized COC [41]. Conversely, it is accepted that a COC/BEG ratio exceeding 0.75 signifies the minimal metabolic conversion of COC to BEG, suggesting the presence of unmetabolized COC (as direct discharge into wastewater systems) [41]. In the present study, COC/BEG ratios spanning from 0.07 to 0.21 were observed in most of the analyzed samples, implying that the principal origin of cocaine (COC) likely stemmed from excretory pathways (urine, feces, or saliva). However, samples WW-04, WW-06, and WW-07 demonstrated substantially elevated COC/BEG ratios exceeding 0.75, suggestive of unconsumed cocaine (Fig. S6). This occurrence may plausibly be attributed to losses incurred during transportation and/or manipulation processes.

Regarding cannabinoids, CBN, CBD, and THC were detected at concentrations ranging from 37 ng L^−1^ to 176 ng L^−1^, from 9.2 ng L^−1^ to 36.5 ng L^−1^, and from 9.6 ng L^−1^ to 29.9 ng L^−1^, respectively. In addition, suspect screening revealed that the THC metabolite 11-hydroxy-Δ9-tetrahydrocannabinol was detected in 60% of the investigated samples, suggesting excretion following cannabis consumption. Cannabis is the most widely consumed illicit drug globally; thus, the presence of traces of these compounds in wastewater systems is justified [2, 58, 62]. The concentrations observed in this study for cannabinoids such as THC were lower than those reported by Campos-Mañas et al. [58], who investigated different types of sample preparation methods for extracting analytes from wastewater containing suspended solids and found THC concentrations ranging from 40 to 160 ng L^−1^. However, it is important to note that the primary objective of this study was to investigate the dissolved fraction, which may contain a reduced amount of these compounds compared to the suspended particulate matter (SPM).

The potent opioid FEN was found in lower concentrations (6.17–14.4 ng L^−1^) than COC, BEG, EME, NOR, and MBDB. This synthetic opioid has been a cause for concern due to its indiscriminate use as an additive in common illegal drugs such as cocaine, heroin, methamphetamine, and MDMA [63, 64]. However, FEN is also used as a pharmaceutical in hospitals for patients receiving intensive care or pain treatment. The detection of additional opioid analgesics by suspect screening, such as tapentadol and tramadol, reinforces the hypothesis of pharmaceutical use.

Amphetamine-type stimulants such as AMP, MDMA, and MBDB were found in only one wastewater sample at concentrations ranging from 12.2 ng L^−1^ to 32.9 ng L^−1^. These drugs are commonly found in clubs and pubs, mainly in large urban centers [4, 65].

**Fig. 6:**
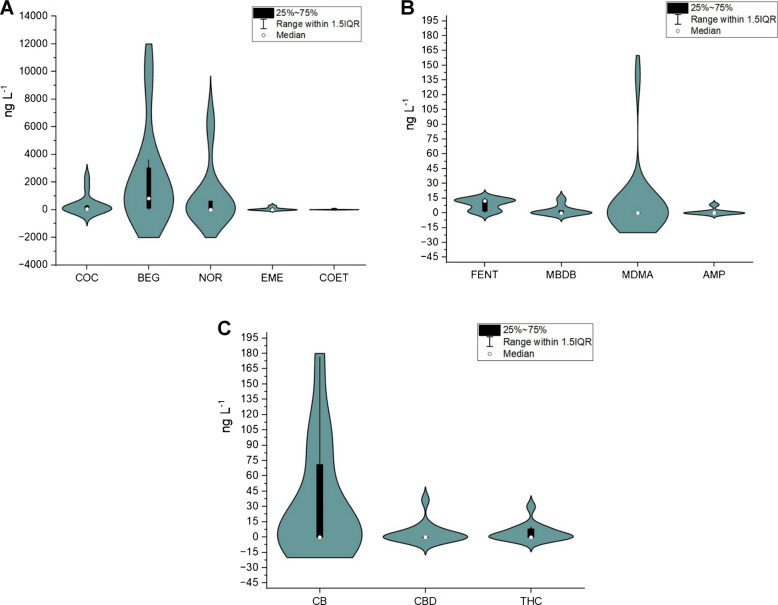
Violin plot graph with Box-Whisker showing the variability of the illicit drug concentrations in the collected wastewater samples. **A** COC and its metabolites. **B** FEN and amphetamine-type stimulants. **C** Cannabinoids

As shown in the violin and box–whisker plots in Fig. 6, COC, BEG, NOR, and EME exhibit marked variability across the sampling sites. The kernel density distribution indicates that, for COC, BEG, and NOR, the highest probability densities are centered near their respective median values, approximately 21 ng L⁻^1^, 142 ng L⁻^1^, and 211 ng L⁻^1^. In contrast, EME, COET, MBDB, and AMP showed comparatively low variability among the analyzed wastewater samples. Regarding cannabinoids, CBN showed greater variability in concentration than CBD and THC, indicating a more heterogeneous distribution across the investigated sites.

### Evaluation of method greenness and comparison of the analytical performance of the developed procedure

**Fig. 7:**
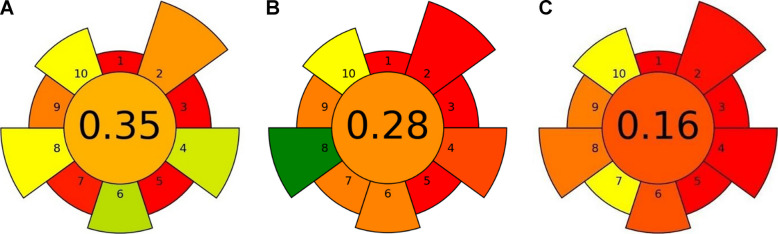
AGREEprep pictogram for the greenness assessment of the developed procedure. **A** Proposed D-µ-SPE method with an overall score of 0.35, **B** conventional SPE using Chromabond HR-X cartridges (200 mg) exhibiting an overall score of 0.28 [8], and **C** automated SPE using Dionex Autotrace AT280 with MCX cartridges, yielding an overall score of 0.16 [4]

The proposed procedure was assessed in accordance with Green Sample Preparation principles, which include: (1) favoring in situ sample preparation; (2) utilizing safer solvents and reagents; (3) employing sustainable, reusable, and renewable materials; (4) minimizing or avoiding waste generation; (5) minimizing sample, chemical and material amounts; (6) maximizing sample throughput; (7) integrating steps and promoting automation; (8) minimizing energy consumption; (9) ensuring green post-sample preparation practices; and (10) guaranteeing operator safety [54]. Each principle was quantitatively evaluated using the AgreePrep metric, which assigns a score ranging from 0 (non-compliant) to 1 (fully compliant) [66]. Based on this assessment, a colored pictogram is generated, displaying each criterion (1 to 10) in the outer circle and the overall score in the center. A score closer to 1 indicates a greener procedure, represented by a green pictogram, whereas a score approaching 0 signifies a less green procedure, depicted by a red pictogram [66]. As shown in Fig. 7A, the proposed procedure reached an overall score of 0.35, considered to exhibit moderate greenness, and therefore requires improvements to be considered of high greenness (> 0.75).

A detailed analysis of the pictogram in Fig. 7 shows that higher scores were obtained for criteria 4 and 6, corresponding to waste minimization and maximization of sample throughput, respectively. In the developed D-µ-SPE method, only 50 mg of sorbent and 1.25 mL of desorption solvent (methanol/5% NH_4_OH) are required, significantly reducing waste. In addition, a sample volume of 100 mL was sufficient to provide an adequate enrichment factor (EF), enabling both suspect screening by HRMS and subsequent quantification of target analytes at ultra-trace levels.

Compared to some conventional solid-phase extraction (SPE) procedures adopted for illicit drug analysis [4, 8] (Fig. 7B and C), the proposed method generates less waste, as it eliminates the need for cartridge conditioning and elution steps and requires a similar sample volume.

Regarding analytical performance, the developed procedure was compared with recently published methods for the extraction of illicit drugs from wastewater, including conventional and automated SPE, and microextraction techniques such as dispersive liquid–liquid microextraction (DLLME) and liquid-phase microextraction (LPME). As shown in Table 3, the proposed procedure achieved RSD and recovery values comparable to those reported for microextraction approaches, including coated blade spray coupled to direct mass spectrometry (CBS-MS), DLLME, and LPME. Notably, the proposed method provided lower LOQ values, which is a key factor for the reliable quantification of illicit drugs in wastewater samples. On the other hand, these microextraction techniques typically require smaller sample volumes, which may be advantageous in situations where the collection of larger sample volumes is not feasible.

**Table 3:** Comparison of the analytical performance of the proposed method with commonly employed extraction techniques for the determination of illicit drugs and pharmaceutical compounds in wastewater, including key validation parameters such as LOD, LOQ, precision (RSD%), and recovery

Extraction technique	Time^a^ (min)	Sample volume (mL)	LOD (ng L^−1^)	LOQ (ng L^−1^)	RSD (%)	Recovery (%)	Ref
D-µ-SPE	30	100	1.40–8.79	4.70–29.3	0.857–6.45	79.3–118	**This work**
SPE	167	500	0.060–533	0.210–1777	8.00–11.0	NI	[35]
CBS-MS	20	1.5	1.50–9.00	5.00–30.0	0.59–9.60	70.7–115	[67]
DLLME	10	10	0.570–14.3	7.00–670	0.300–11.1	NI	[39]
SPE	NI	1000	4.00 × 10^−5^–5.00 × 10^−3^	3.00 × 10^−4^–2.81 × 10^−2^	NI	NI	[8]
SPE	NI	100	0.100–26.5	0.600–52.9	2.70–10.9	39.0–166	[68]
SPE*	NI	200	NI	NI	NI	NI	[4]
SPE	16	50	0.330	1.00	0.900–7.40	95.0–120	[69]
LPME	30	12	12.0–20.0	39.9–73.3	6.00–11.3	80.4–114	[12]

^a^Extraction duration. *N*I, not informed. *This work employed the same method reported by Baker et al. [62]

Overall, SPE methods employing sample volumes greater than 100 mL exhibit lower LOD and LOQ values than the proposed D-µ-SPE approach, while providing comparable RSD values (Table 3). This trend can be attributed to the increase in enrichment factor associated with larger sample volumes, which contributes to improved sensitivity. Importantly, the proposed method in this work employed a fixed sample volume of 100 mL, which was compatible with the customized glass device developed for D-µ-SPE extractions and did not compromise the LOQ values.

Additionally, the proposed procedure enabled sensitive detection in both target and suspect screening of illicit drugs and a broad range of pharmaceutical substances, with high-confidence confirmation achieved by high-resolution mass spectrometry and comparison with analytical standards. These characteristics indicate that the method is suitable for routine and environmental surveillance of these compounds in wastewater. Nevertheless, the relatively low extraction recoveries observed for certain cannabinoids, together with the extraction time of 30 min, constitute the main limitations of the methodology.

## Conclusion

An efficient D-µ-SPE analytical method was successfully developed in combination with the UHPLC-QTOF-MS and UHPLC-QqQ-MS systems for both suspect screening and highly sensitive quantification of illicit drugs and pharmaceutical substances in complex wastewater samples.

The method provided adequate enrichment factors and extraction recoveries using only 100 mL of sample, eliminated solvent evaporation steps, and enabled sequential suspect screening and multi-analyte determination within a single sample preparation workflow. In comparison with other extraction methodologies, the proposed approach exhibited comparable LOD, LOQ, RSD, and recovery values. However, the use of a fixed sample volume of 100 mL may limit sensitivity when compared with traditional SPE methodologies that typically employ larger sample volumes.

The Analytical Greenness Metric for Sample Preparation (AGREEprep) was applied to assess the greenness of the developed method. Although the D-µ-SPE method showed some limitations (overall score < 0.5), it achieved favorable scores for minimizing or avoiding waste generation and maximizing sample throughput.

The application of the proposed method to real wastewater samples enabled suspect screening using data-dependent acquisition, allowing the identification of potential illicit drugs, metabolites, and pharmaceutical substances. Central nervous system stimulants, opioid analgesics, cardiovascular drugs, and cannabis-related biomarkers were detected. Quantification of target analytes by UHPLC-QqQ-MS confirmed the presence of illicit drugs such as cocaine and its metabolites, as well as synthetic amphetamine derivatives, including MDMA and MBDB. Cannabinoids were also detected, and some were quantified. The presence of the metabolite 11-hydroxy-THC suggested excretion as the primary pathway by which these substances enter wastewater.

Although lower recoveries were observed for selected cannabinoids and the extraction time remains moderate, the overall robustness, analytical scope, and compatibility with retrospective HRMS data analysis make this workflow a promising tool for routine environmental and epidemiological monitoring.

## Supplementary Information

Below is the link to the electronic supplementary material.

Supplementary file1 (DOCX 2.30 MB)

## Data Availability

The authors affirm that the data supporting the study’s findings are accessible within the paper and its Supplementary Information files. If raw data files are required in a different format, they can be obtained from the corresponding author upon reasonable request.
