# Supplementary material for: Dispersive micro-solid-phase extraction coupled to UHPLC-Q-TOF-MS and UHPLC-QqQ-MS for suspect and target screening of illicit drugs and pharmaceuticals in wastewater
Source: Anal Bioanal Chem. 2026 Apr 28;418(13):4233–52. doi: 10.1007/s00216-026-06520-3 (PMC13264539; doi:10.1007/s00216-026-06520-3)
Supplement: Supplementary file 1 — Supplementary file1 (DOCX 2.30 MB) [file 216_2026_6520_MOESM1_ESM.docx]

**SUPPLEMENTARY MATERIAL**

**Dispersive micro-solid-phase extraction coupled to UHPLC-Q-TOF-MS and UHPLC-QqQ-MS for suspect and target screening of illicit drugs and pharmaceuticals in wastewater**

Madson M. Nascimento*^a,e^, Paulo R. R. Mesquita^b^, Ricardo L. Cunha^c^, André L. S. da Silva Junior^f^, Gisele O. da Rocha^d,e^, Raildo M. de Jesus^f^, Pedro Afonso de P. Pereira^d,e^, Jailson B. de Andrade^a,e^

^a^Universidade SENAI CIMATEC, Av. Orlando Gomes, 1845 - Piatã, 41650-010, Salvador – BA, Brazil.

^b^Centro Tecnológico Agropecuário do Estado da Bahia - CETAB, Secretaria da Agricultura, Pecuária, Irrigação, Pesca e Aquicultura – SEAGRI Av. Milton Santos, 967 - Ondina, Salvador - BA, 40170-110.

^c^Laboratório de Toxicologia Forense, Instituto de Análises e Pesquisas Forenses – IAPF, Polícia Científica, São Cristóvão, SE 49100-000, Brazil.

^d^Universidade Federal da Bahia, Instituto de Química, Campus de Ondina, 40170-115 Salvador, BA, Brazil.

^e^Instituto Nacional de Ciência e Tecnologia em Energia e Ambiente - INCT, Universidade Federal da Bahia, 40170-115 Salvador, BA, Brazil.

^f^Universidade Estadual de Santa Cruz, Campus Soane Nazaré de Andrade, Rod. Jorge Amado, Km 16 - Salobrinho, Ilhéus - BA, 45662-900

André L. S. da Silva Junior ORCID ID: 0000-0002-8508-7007

Gisele Olimpio da Rocha ORCID ID: 0000-0002-1847-8745

Jailson Bittencourt de Andrade ORCID ID: 0000-0001-5269-4866

Madson Moreira Nascimento ORCID ID: 0000-0003-4405-6519

Paulo R. R. Mesquita ORCID ID: 0000-0003-3187-3800

Pedro Afonso de Paula Pereira ORCID ID: B-6223-2014

Raildo M. de Jesus ORCID ID: 0000-0001-6366-948X

Ricardo Cunha Leal OCRID ID: 0000-0003-0444-7742

∗Corresponding author: Madson M. Nascimento

E-mail address: madchemis89@gmail.com

1. **Chromatographic separation and selection of the mobile phase**

The initial separation tests used the organic phase of 0.1% formic acid in water (A) and pure methanol (D). Different gradient programming was investigated, and the best result, in terms of signal intensity, was achieved by initiating elution with 95% A and 5% B at time 0 min, increasing to 85% B within 7 min, and reaching 100% B at 15 min, maintaining this percentage until 16 min. The best flow rate was 0.4 mL min^-1^. The chromatographic peaks generally exhibit good separation and symmetric shape (Fig. S1-3). The two most intense fragments for each compound were selected to set up the transitions of the dMRM method (Table S1).

After optimizing the separation of target analytes, we performed the mobile phase composition study. The effectiveness of the separation and the ionization of ionizable analytes, such as drugs or illicit drugs, relies on using an appropriate mobile phase in liquid chromatography coupled with mass spectrometry techniques. Incorporating chemical modifiers in the mobile phase becomes imperative when utilizing Electrospray Ionization (ESI) sources to augment the ionization efficiency of target analytes and, consequently, enhance the sensitivity of the chromatographic method.

As depicted in Fig. S4, the organic mobile phase, which was methanol with 0.5 mM ammonium formate (as a chemical modifier) yielded superior results (average peak area, n=4) for most target analytes when employing the conventional ESI source in positive mode. Experiments conducted under 0.1% formic acid conditions in methanol and acetonitrile did not yield satisfactory improvements in peak areas. One plausible explanation is the low ionic strength of formic acid (0.2 M formic acid; ionic strength 1.9 mM) compared to ammonium formate (Johnson et al., 2013; McCalley, 2004). Using a buffer solution composed of 0.1% formic acid/0.5 mM ammonium formate as a mobile phase also increased peak areas. However, the results obtained with 0.5 mM ammonium formate in methanol were the best ones. Consequently, this mobile phase composition was selected for subsequent steps.

1. Method validation

The ME was evaluated through post extraction addition protocol by comparing the peak area of the target analytes in the standard solution at 50 ng mL^-1^ in pure methanol (A) to that of a postextraction spiked wastewater analyte-free sample (B) at the same concentration. The percentage of ME was calculated according to Eq. 1 (Wu et al., 2022):

ME (%) = $\left( \frac{B}{A}-1 \right)$ x 100 % (1)

The following criteria were applied: if ME = 0, there is no evidence of matrix effect. On the other hand, if ME is higher or lower than 0, there is evidence of signal enhancement or ion suppression (Wu et al., 2022).

The linear range and linearity were evaluated by an external matrix-matched analytical curve containing ten concentration levels, which ranged from 0.5 to 500 ng L^-1^ from synthetic cathinones, amphetamines, cocaine, and metabolites, and from 2.5 to 2,000 ng L^-1^ for cannabinoids. Each concentration level was extracted using the proposed procedure through 100 mL of the standard solution diluted in analyte-free wastewater. The ANOVA (p<0.05) was used to evaluate the significance of the linear model in all analytical curves. In addition, the appropriateness of the determination coefficient (R^2^) was assessed by employing the *t*-student test (Eq. 2) (Da Silva et al., 2019). All analytical curves were verified for homoscedasticity.

t_cal_ = $\sqrt{R^{2}}$ $\sqrt{(n-2)/(1-R^{2})}$ (2)

The limit of detection (LOD) and limit of quantification (LOQ) were calculated through analytical curve parameters (Ribani et al., 2007), as expressed in Eq. 3 and 4:

LOD = $\frac{3 x SB}{a}$ (3)

LOQ = $\frac{10 x SB}{a}$ (4)

Where “SB” is the standard deviation of the linear coefficient and “a” is the slope of the matrix-matched analytical curve.

The precision was assessed as repeatability and intermediate precision. The repeatability was obtained by calculating ten independent extractions' relative standard deviation (RSD) using the D-µ-SPE procedure in a single day (n=10). On the other hand, the intermediate precision was calculated by performing ten extractions in three consecutive days (n=30).

The accuracy of the analytical procedure was evaluated through relative recoveries at three different concentration levels. A set of wastewater samples and river water samples were spiked at 25, 50, and 100 ng L^-1^ for amphetamines, synthetic cathinones, cocaine, and metabolites, 200, 400, and 800 ng L^-1^ for cannabinoids. A volume of 100 mL of these spiked samples was extracted in triplicate using the proposed D-µ-SPE procedure. The relative recovery (RR) was calculated according to the Eq. 5:

RR = $\left( \frac{Concentration quantified in spiked sample- Concentration in unspiked sample}{Concentration added (true value)} \right)$ x 100 (5)

The extraction efficiency assessment involved utilizing the enrichment factor (EF) and the extraction recovery (ER), both determined through the mathematical expressions presented in Eq. 6 and 7, respectively.

EF = $\left( \frac{A organic}{A aqueous} \right)$ (6)

ER = $\left( \frac{n organic}{n aqueous} \right)$ × 100 % = $\left( \frac{V organic}{V aqueous} \right)$ × $\left( \frac{A organic}{A aqueous} \right)$ × 100 (7)

The variable "*A organic*" represents the concentration of the target analytes determined in the organic solvent after sorbent desorption, while "*A aqueous*" denotes the initial concentrations of the target analytes in the wastewater sample. "*n organic*" correlates to the quantity of target analytes concentrated in the desorption solvent after to the D-µ-SPE procedure, whereas "*n aqueous*" signifies the total amount of the target analytes initially present in the wastewater sample. Additionally, "*V organic*" and "*V aqueous*" stands for the volumes of the extractor solvent and the sample solution, respectively (Nojavan & Yazdanpanah, 2017).

Table S1. Retention time and UHPLC-QqQ-MS parameters for the determination of target illicit drugs.

| **Compounds** | **Transition** | **Type** | **Precursor Ion** | **Product Ion** | **RT** | **Ion Polarity** | **Collision Energy** | **Fragmentor (V)** |
| --- | --- | --- | --- | --- | --- | --- | --- | --- |
| Norfentanyl-d_5_ | 238.0 -> 155.0 | Target | 238.0 | 155.0 | 6.03 | Positive | 25 | 100 |
| Norfentanyl-d_5_ | 238.0 -> 137.2 | Qualifier |  | 137.2 |  | Positive | 25 | 100 |
| Norcocaine | 290.0 -> 168.0 | Target | 290.0 | 168.0 | 6.50 | Positive | 13 | 100 |
| Norcocaine | 290.0 -> 136 | Qualifier |  | 136 |  | Positive | 13 | 100 |
| N-ethylpentylone | 250.0 -> 202.0 | Target | 250.0 | 202.0 | 6.50 | Positive | 25 | 80 |
| N-ethylpentylone | 250.0 -> 231.9 | Qualifier |  | 231.9 |  | Positive | 25 | 80 |
| Metilone | 208.0 -> 159.9 | Target | 208.0 | 159.9 | 4.92 | Positive | 20 | 80 |
| Metilone | 208.0 -> 132 | Qualifier |  | 132 |  | Positive | 20 | 80 |
| Metanphetamine-d_11_ | 161.0 -> 127.0 | Target | 161.0 | 127.0 | 5.28 | Positive | 20 | 100 |
| Metanphetamine-d_11_ | 161.0 -> 108 | Qualifier |  | 108 |  | Positive | 20 | 100 |
| Metanphetamine | 150.0 -> 91.1 | Target | 150.0 | 91.1 | 5.34 | Positive | 15 | 80 |
| Metanphetamine | 150.0 -> 119 | Qualifier |  | 119 |  | Positive | 15 | 80 |
| MDMA-d_5_ | 199.1 -> 165.1 | Target | 199.1 | 165.1 | 5.36 | Positive | 13 | 100 |
| MDMA-d_5_ | 199.1 -> 107.1 | Qualifier |  | 107.1 |  | Positive | 13 | 100 |
| MDMA | 194.0 -> 134.9 | Target | 194.0 | 134.9 | 5.38 | Positive | 15 | 80 |
| MDMA | 194.0 -> 104.8 | Qualifier |  | 104.8 |  | Positive | 15 | 80 |
| MDEA | 208.0 -> 162.9 | Target | 208.0 | 162.9 | 5.66 | Positive | 15 | 80 |
| MDEA | 208.0 -> 105 | Qualifier |  | 105 |  | Positive | 15 | 80 |
| MDA | 180.0 -> 105.1 | Target | 180.0 | 105.1 | 5.50 | Positive | 5 | 80 |
| MDA | 180.0 -> 162.9 | Qualifier |  | 162.9 |  | Positive | 5 | 80 |
| MBDB | 208.0 -> 135.0 | Target | 208.0 | 135.0 | 5.66 | Positive | 15 | 80 |
| MBDB | 208.0 -> 146.9 | Qualifier |  | 146.9 |  | Positive | 15 | 80 |
| Fentanyl | 337.0 -> 188.1 | Target | 337.0 | 188.1 | 7.10 | Positive | 25 | 80 |
| Fentanyl | 337.0 -> 104.9 | Qualifier |  | 104.9 |  | Positive | 25 | 80 |
| Fenproporex | 189.0 -> 90.9 | Target | 189.0 | 90.9 | 5.23 | Positive | 21 | 70 |
| Fenproporex | 189.0 -> 188.1 | Qualifier |  | 188.1 |  | Positive | 21 | 70 |
| Etilone | 222.0 -> 174.0 | Target | 222.0 | 174.0 | 5.24 | Positive | 20 | 80 |
| Etilone | 222.0 -> 203.8 | Qualifier |  | 203.8 |  | Positive | 20 | 80 |
| Ecgononine methyl ester | 200.0 -> 81.9 | Target | 200.0 | 81.9 | 1.43 | Positive | 30 | 80 |
| Ecgononine methyl ester | 200.0 -> 182 | Qualifier |  | 182 |  | Positive | 30 | 80 |
| Dronabinol-THC | 315.0 -> 259.1 | Target | 315.0 | 259.1 | 13.24 | Positive | 25 | 80 |
| Dronabinol-THC | 315.0 -> 193.1 | Qualifier |  | 193.1 |  | Positive | 25 | 80 |
| Delta-9-THC-d_3_ | 318.0 -> 196.0 | Target | 318.0 | 196.0 | 12.91 | Positive | 25 | 100 |
| Delta-9-THC-d_3_ | 318.0 -> 135 | Qualifier |  | 135 |  | Positive | 25 | 100 |
| Cocaine-d_3_ | 307.0 -> 185.0 | Target | 307.0 | 185.0 | 6.25 | Positive | 20 | 100 |
| Cocaine-d_3_ | 307.0 -> 153 | Qualifier |  | 153 |  | Positive | 20 | 100 |
| Cocaine | 304.0 -> 182.0 | Target | 304.0 | 182.0 | 6.25 | Positive | 25 | 80 |
| Cocaine | 304.0 -> 150 | Qualifier |  | 150 |  | Positive | 25 | 80 |
| Cocaethylene | 318.0 -> 195.9 | Target | 318.0 | 195.9 | 6.77 | Positive | 20 | 80 |
| Cocaethylene | 318.0 -> 150 | Qualifier |  | 150 |  | Positive | 20 | 80 |
| Clobenzorex | 260.0 -> 90.9 | Target | 260.0 | 90.9 | 7.57 | Positive | 20 | 80 |
| Clobenzorex | 260.0 -> 119.1 | Qualifier |  | 119.1 |  | Positive | 20 | 80 |
| Cannabinol | 311.0 -> 222.9 | Target | 311.0 | 222.9 | 12.56 | Positive | 21 | 125 |
| Cannabinol | 311.0 -> 195 | Qualifier |  | 195 |  | Positive | 21 | 125 |
| Cannabidiol | 315.0 -> 123.1 | Target | 315.0 | 123.1 | 11.09 | Positive | 30 | 100 |
| Cannabidiol | 315.0 -> 193.1 | Qualifier |  | 193.1 |  | Positive | 30 | 100 |
| Benzoylecgnonine | 290.0 -> 167.9 | Target | 290.0 | 167.9 | 6.06 | Positive | 20 | 80 |
| Benzoylecgnonine | 290.0 -> 272.3 | Qualifier |  | 272.3 |  | Positive | 20 | 80 |
| Amphetamine-d_6_ | 142.0 -> 93.1 | Target | 142.0 | 93.1 | 5.22 | Positive | 13 | 100 |
| Amphetamine-d_6_ | 142.0 -> 125 | Qualifier |  | 125 |  | Positive | 13 | 100 |
| Amphetamine | 136.0 -> 91.0 | Target | 136.0 | 91.0 | 5.25 | Positive | 17 | 60 |
| Amphetamine | 136.0 -> 90.8 | Qualifier |  | 90.8 |  | Positive | 17 | 60 |

Table S2. Retention time and identification of the target analytes by UHPLC-QTOF-MS.

| **Compound**  **Name** | **Molecular Formula** | **Rt (min)** | **Molecular weight** | **Theoretical *m/z***  **[M + H]^+^** | **Measured *m/z***  **[M + H]^+^** | **Mass error (ppm)** |
| --- | --- | --- | --- | --- | --- | --- |
| AMP | C_9_H_13_N | 3.93 | 135.1048 | 136.1121 | 136.1117 | -2.77 |
| BEG | C_16_H_19_NO_4_ | 4.65 | 289.1314 | 290.1387 | 290.1392 | 1.80 |
| CBD | C_21_H_30_O_2_ | 9.57 | 314.2246 | 315.2319 | 315.2323 | 1.34 |
| CBN | C_21_H_26_O_2_ | 10.83 | 310.1933 | 311.2006 | 311.2010 | 1.36 |
| CBZ | C_16_H_18_ClN | 6.13 | 259.1128 | 260.1201 | 260.1201 | 0.09 |
| COC | C_17_H_21_NO_4_ | 4.99 | 303.1470 | 304.1543 | 304.1550 | 2.30 |
| COET | C_18_H_23_NO_4_ | 5.33 | 317.1627 | 318.1700 | 318.1711 | 3.53 |
| EME | C_10_H_17_NO_3_ | 1.26 | 199.1208 | 200.1281 | 200.1284 | -1.61 |
| ETH | C_12_H_15_NO_3_ | 3.88 | 221.1052 | 222.1125 | 222.1124 | 0.35 |
| FEN | C_12_H_16_N_2_ | 3.86 | 188.1313 | 189.1386 | 189.1388 | -1.18 |
| FENT | C_22_H_28_N_2_O | 5.77 | 336.2202 | 337.2275 | 337.2281 | 1.85 |
| MBDB | C_12_H_17_NO_2_ | 4.61 | 207.1259 | 208.1332 | 208.1326 | -2.77 |
| MDA | C_10_H_13_NO_2_ | 3.95 | 179.0946 | 180.1019 | 180.1024 | -2.90 |
| MDEA | C_12_H_17_NO_2_ | 4.22 | 207.1259 | 208.1332 | 208.1341 | 4.43 |
| MDMA | C_11_H_15_NO_2_ | 4.01 | 193.1103 | 194.1176 | 194.1176 | 0.12 |
| MET | C_11_H_13_NO_3_ | 3.57 | 207.0895 | 208.0968 | 208.0970 | -1.07 |
| METH | C_10_H_15_N | 4.02 | 149.1204 | 150.1277 | 150.1282 | -3.48 |
| NEP | C_14_H_19_NO_3_ | 5.11 | 249.1365 | 250.1438 | 250.1444 | 2.49 |
| NOR | C_16_H_19_NO_4_ | 5.05 | 289.1314 | 290.1387 | 290.1376 | -3.79 |
| THC | C_21_H_30_O_2_ | 11.44 | 314.2246 | 315.2319 | 315.2325 | 1.98 |

The initial approach consisted of separating and identifying the target compounds by LC-QTOF-MS through the injection of authentic standards. At this stage, a mixed solution containing the analytes at a concentration of 5 mg L^-1^ was injected into the UHPLC-QTOF-MS in MS mode. Information regarding retention times, molecular formulas, and exact mass is shown in Table 3. The results indicated that it was possible to identify all compounds with a mass error below 5 ppm, being suitable for screening potential illicit drugs and their metabolites.

Table S3. Doehlert matrix design for three variables (A) and (B) ANOVA (p<0.05) for validation of the obtained quadratic model.

**A)**

|  | **MCX (mg)** | **NaCl (%)** | **Extraction time (min)** | **MR** |
| --- | --- | --- | --- | --- |
| 1 | 0 (50) | 0 (7.5) | 0 (30) | 10.8 |
| 2 | 1 (70) | 0 (7.5) | 0 (30) | 10.6 |
| 3 | 0.5 (60) | 0.866 (10) | 0 (30) | 11.5 |
| 4 | 0.5 (60) | 0.289 (8.3) | 0.817 (35) | 11.5 |
| 5 | -1 (30) | 0 (7.5) | 0 (30) | 11.0 |
| 6 | -0.5(40) | -0.866 (5) | 0 (30) | 11.3 |
| 7 | -0.5(40) | -0.289 (5.8) | -0.817 (25) | 10.5 |
| 8 | 0.5 (60) | -0.866 (5) | 0 (30) | 10.5 |
| 9 | 0.5 (60) | -0.289 (5.8) | -0.817 (25) | 10.2 |
| 10 | -0.5(40) | 0.866 (10) | 0 (30) | 10.7 |
| 11 | 0 (50) | 0.577 (9.2) | -0.817 (25) | 11.3 |
| 12 | -0.5(40) | 0.289(8.3) | 0.817 (35) | 12.2 |
| 13 | 0(50) | -0.577 (5.1) | 0.817 (35) | 10.7 |
| 14 | 0(50) | 0 (7.5) | 0 (30) | 11.5 |
| 15 | 0(50) | 0 (7.5) | 0 (30) | 11.6 |
| 16 | 0(50) | 0 (7.5) | 0 (30) | 11.9 |

**B)**

| **Source of variation** | **SS** | **df** | **MS** | **F** | **F-critical** |
| --- | --- | --- | --- | --- | --- |
| MSR | 3.48 | 9 | 0.39 | 1.39 | 4.10 |
| MSr | 1.67 | 6 | 0.28 |  |  |
| Lack of Fit | 0.487 | 3 | 0.244 | 1.11 | 9.28 |
| Pure Error | 0.659 | 3 | 0.220 |  |  |

SS. Sum of squares. df. Degree of freedom. MS. Mean square. F. Calculated F-value. MCX (mg). Amount of MCX sorbent. NaCl(%). Percent (wt.) of sodium chloride.

Table S4. Average relative recoveries (n=3, ±RSD) obtained from spiked wastewater and river water samples.

| **Recovery (%) in wastewater** | | | | | | | | | |  | **Recovery (%) in riverine water** | | | | | | |
| --- | --- | --- | --- | --- | --- | --- | --- | --- | --- | --- | --- | --- | --- | --- | --- | --- | --- |
| Compounds | Low level | | | Medium level | | | High level | | |  | Low level | | | | Medium level | | |
|  | 25 ng L^-1^ | | | 50 ng L^-1^ | | | 100 ng L^-1^ | | |  | 25 ng L^-1^ | | | | 50 ng L^-1^ | | |
| AMP | 95.3 | ± | 0.7 | 92.1 | ± | 0.7 | 94.1 | ± | 1.1 |  | 105 | ± | 4 | 99.6 | | ± | 0.2 |
| BEG | 105 | ± | 6 | 103 | ± | 7 | 114 | ± | 3 |  | 60.7 | ± | 9.7 | 97.0 | | ± | 3.9 |
| CBZ | 84.1 | ± | 0.8 | 83.4 | ± | 1.0 | 83.7 | ± | 1.3 |  | 88.3 | ± | 0.7 | 88.0 | | ± | 2.4 |
| COC | 108 | ± | 2 | 106 | ± | 1 | 109 | ± | 1 |  | 105 | ± | 1 | 98.0 | | ± | 0.5 |
| COET | 103 | ± | 1 | 102 | ± | 0 | 105 | ± | 0 |  | 103 | ± | 2 | 97.6 | | ± | 1.3 |
| EME | 101 | ± | 5.9 | 83.7 | ± | 2.1 | 70.9 | ± | 2.5 |  | 89.7 | ± | 1.4 | 90.6 | | ± | 10.0 |
| ETH | 95.3 | ± | 0.7 | 92.1 | ± | 0.7 | 94.1 | ± | 1.1 |  | 94.2 | ± | 0.3 | 91.1 | | ± | 2.6 |
| FEN | 80.7 | ± | 0.7 | 79.3 | ± | 0.9 | 82.5 | ± | 1.2 |  | 92.0 | ± | 4.2 | 88.7 | | ± | 3.8 |
| FENT | 94.8 | ± | 1.9 | 91.5 | ± | 1.6 | 91.7 | ± | 0.7 |  | 104 | ± | 1 | 101 | | ± | 0 |
| MDA | 115 | ± | 1.2 | 108 | ± | 4.5 | 89.7 | ± | 1.5 |  | 105 | ± | 18 | 80.1 | | ± | 1.1 |
| MBDB | 92.6 | ± | 1.5 | 92.0 | ± | 1.8 | 93.5 | ± | 2.3 |  | 102 | ± | 1 | 99.3 | | ± | 2.1 |
| MDEA | 87.7 | ± | 1.1 | 88.3 | ± | 0.8 | 91.4 | ± | 0.4 |  | 96.9 | ± | 1.6 | 93.5 | | ± | 0.5 |
| MDMA | 81.6 | ± | 6.9 | 81.1 | ± | 0.3 | 89.4 | ± | 2.0 |  | 102 | ± | 15 | 97.9 | | ± | 8.2 |
| MET | 84.7 | ± | 1.2 | 84.4 | ± | 0.3 | 85.0 | ± | 0.5 |  | 100 | ± | 1 | 93.0 | | ± | 1.0 |
| METH | 90.9 | ± | 1.2 | 90.7 | ± | 0.8 | 93.8 | ± | 0.4 |  | 104 | ± | 1 | 99.4 | | ± | 0.5 |
| NEP | 97.0 | ± | 0.5 | 94.9 | ± | 0.2 | 95.6 | ± | 0.8 |  | 103 | ± | 1 | 98.9 | | ± | 0.6 |
| NOR | 118 | ± | 1.8 | 113 | ± | 2.3 | 113.3 | ± | 1.3 |  | 105 | ± | 3 | 94.3 | | ± | 2.1 |
|  | Cannabinoids | | | | | | | | |  | Cannabinoids | | | | | | |
| Compounds | 400 ng L^-1^ | | | 600 ng L^-1^ | | | 2,000 ng L^-1^ | | |  | 400 ng L^-1^ | | | | 600 ng L^-1^ | | |
| THC | 88.1 | ± | 3.7 | 109 | ± | 3.1 | 86.0 | ± | 2.3 |  | 101 | ± | 1 | 110 | | ± | 1 |
| CBN | 89.3 | ± | 0.8 | 101 | ± | 4.0 | 84.7 | ± | 1.9 |  | 105 | ± | 2 | 118 | | ± | 1 |
| CBD | 89.3 | ± | 0.8 | 113 | ± | 0.5 | 92.4 | ± | 0.5 |  | 105 | ± | 2 | 118 | | ± | 1 |


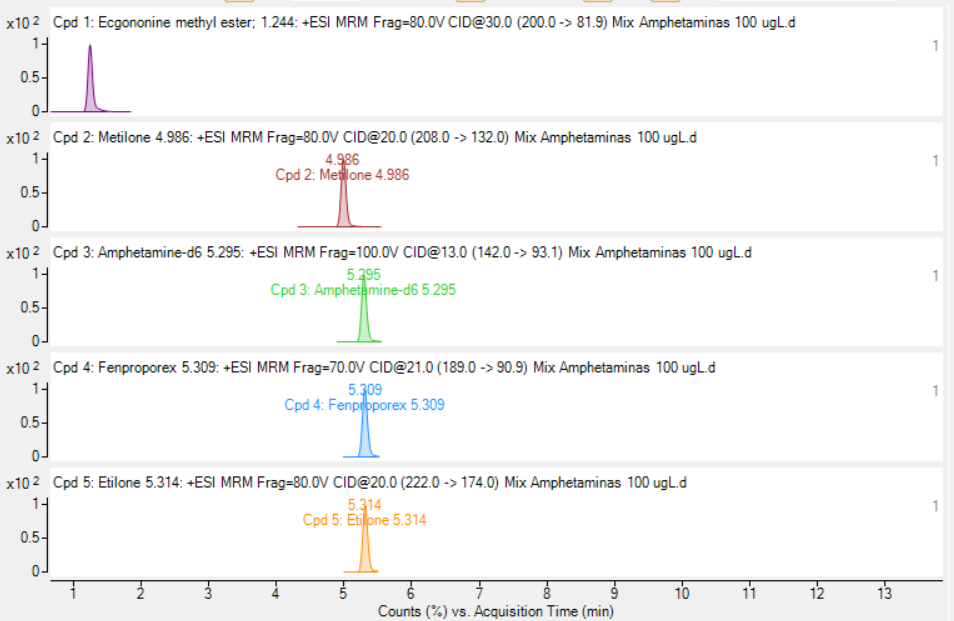


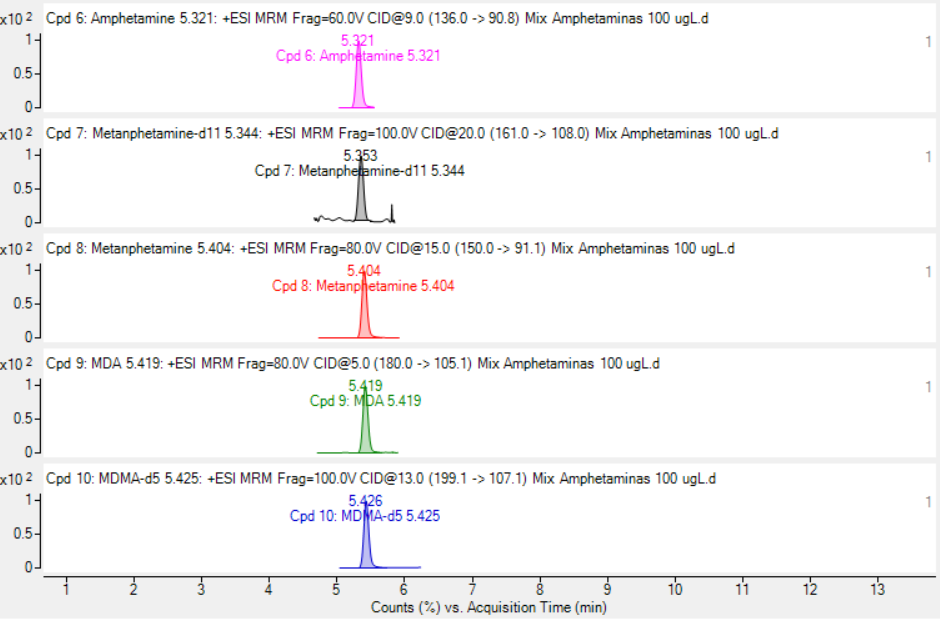


Fig. S1. Dynamic multiple reacting monitoring d-MRM LC-MS/MS chromatogram for the studied illicit drugs. The target analytes are listed by elution order: [1] ecgonine methyl ester, [2] methylone, [3] amphetamine-d_6_, [4] fenproporex, [5] ethylone, [6] amphetamine, [7] methamphetamine-d_11_, [8] metamphetamine, [9] MDA, and [10] MDMA-d_5_, [11].


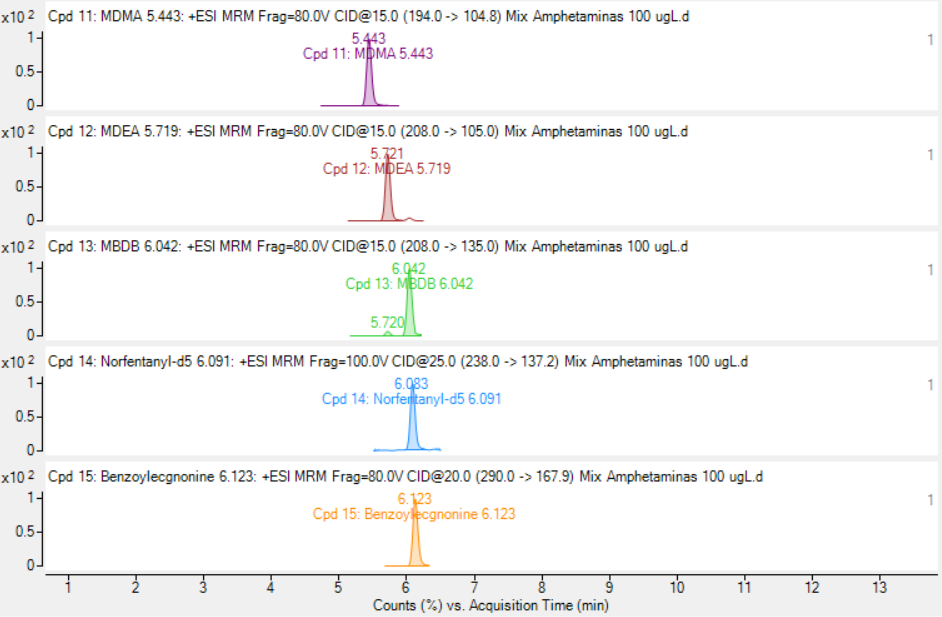


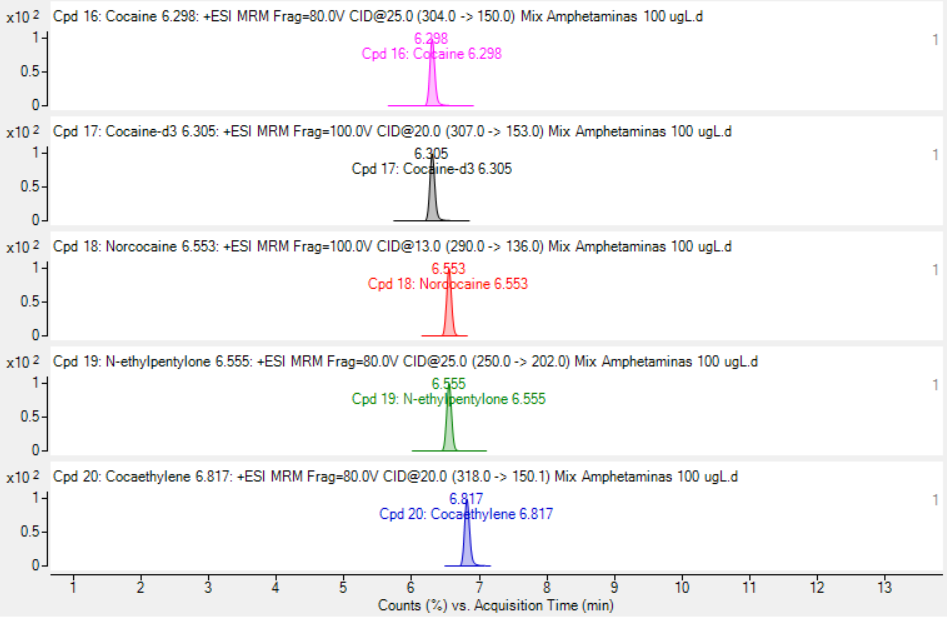


Fig. S2. Dynamic multiple reacting monitoring d-MRM LC-MS/MS chromatogram for the studied illicit drugs. The target analytes are listed by elution order: [12] MDMA, [13] MDEA, [14] MBDB, [15] norfentanyl-d_5_, [16] benzoylecgonine, [17] cocaine, [18] cocaine-d_3_, [19] norcocaine, [20] N-ethylpentylone, [21] cocaethylene.


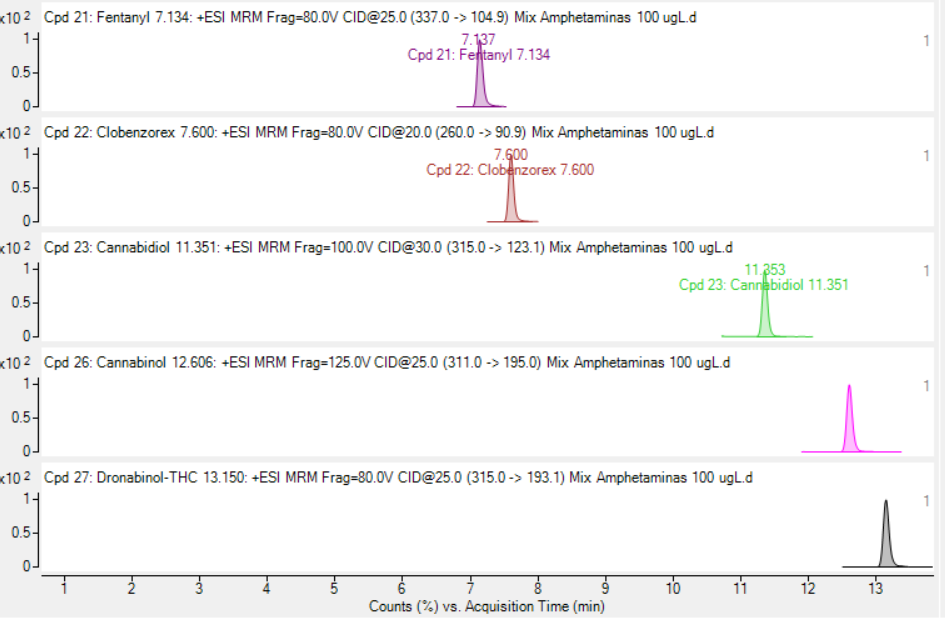


Fig. S3. Dynamic multiple reacting monitoring d-MRM LC-MS/MS chromatogram for the studied illicit drugs. The target analytes are listed by elution order: [22] fentanyl, [23] clobenzorex, [24] cannabidiol, [25] cannabinol, [27] THC.


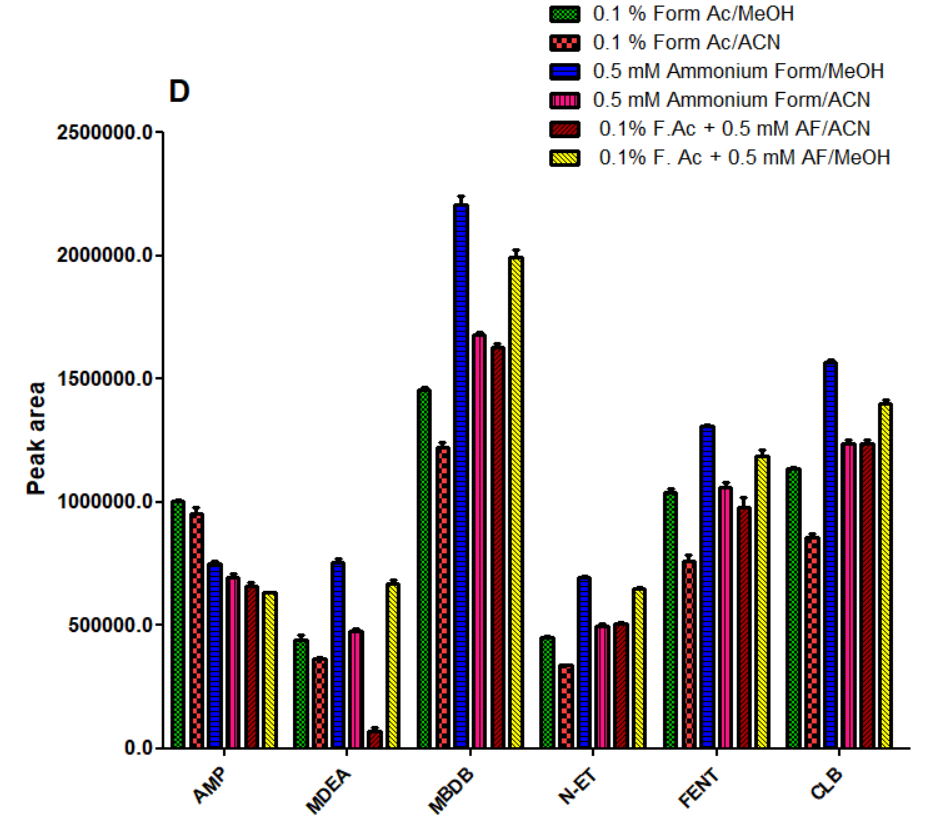

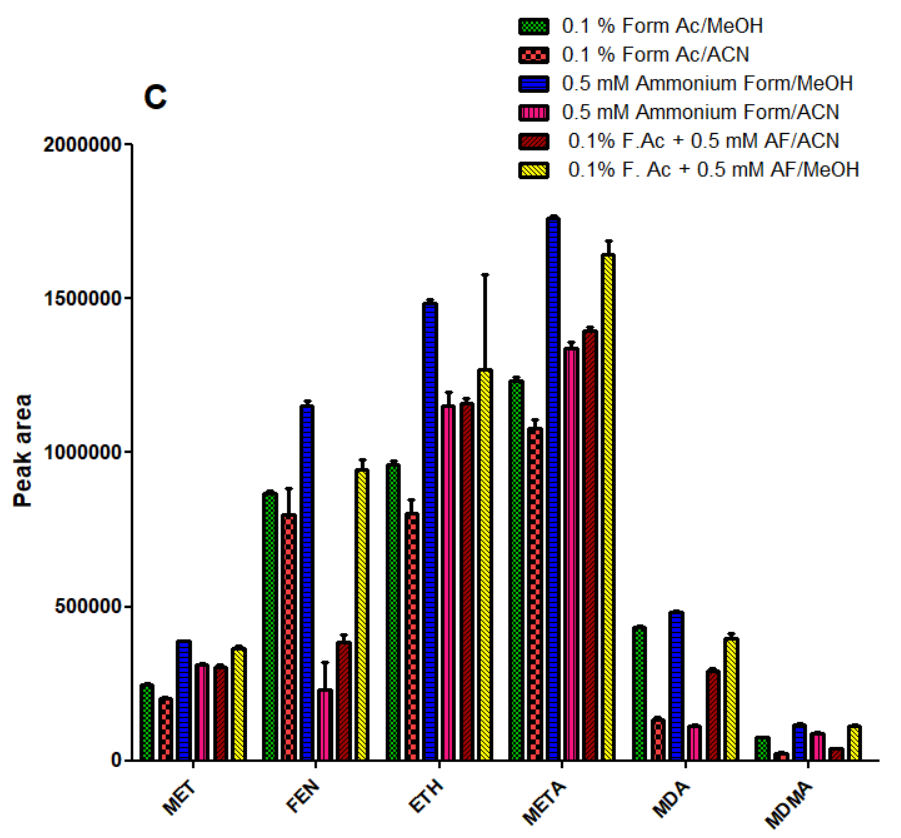

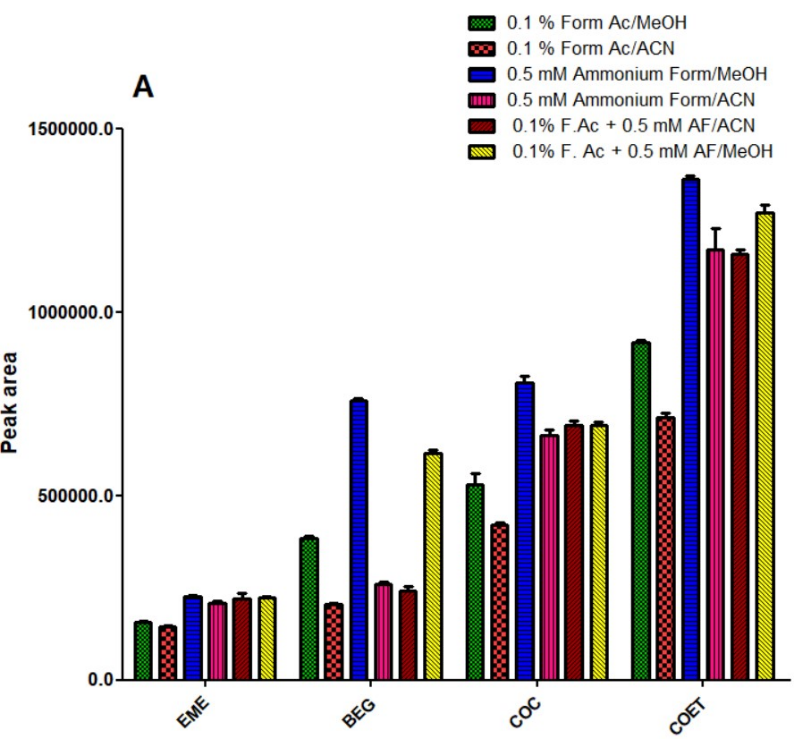

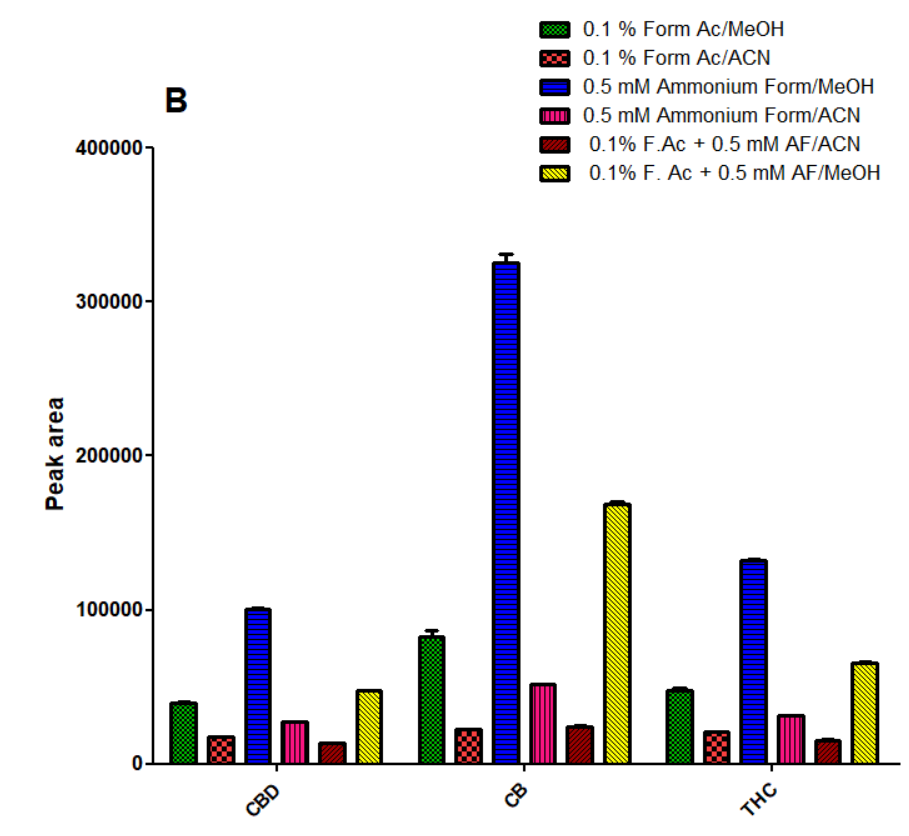


Fig. S4. Study of the effect of the mobile phase on analytical response of A) cocaine and metabolites, B) cannabinoids and its metabolites, and C) synthetic amphetamines and cathinones.


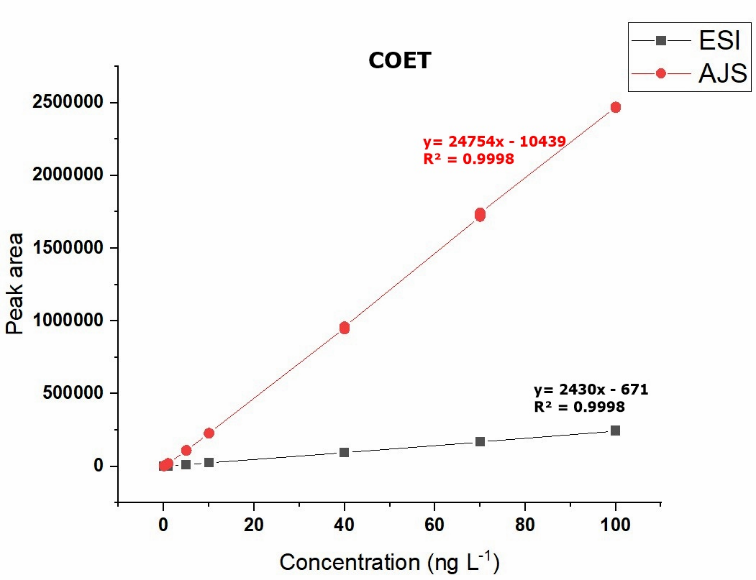

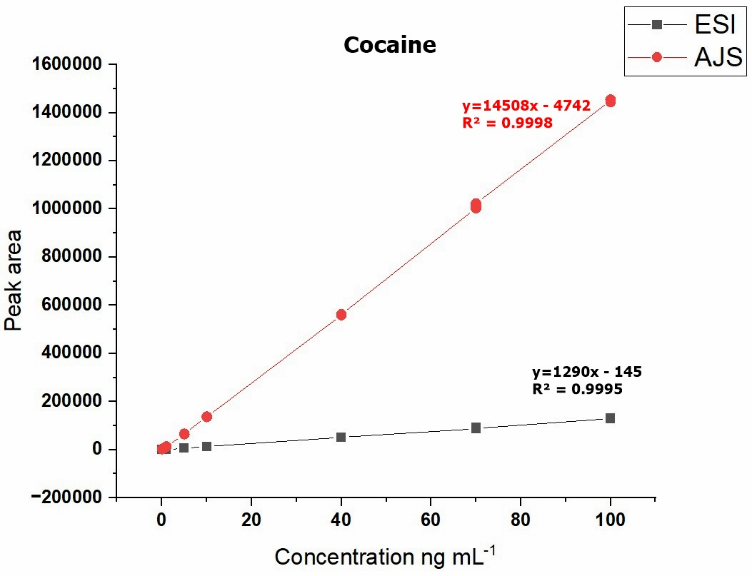

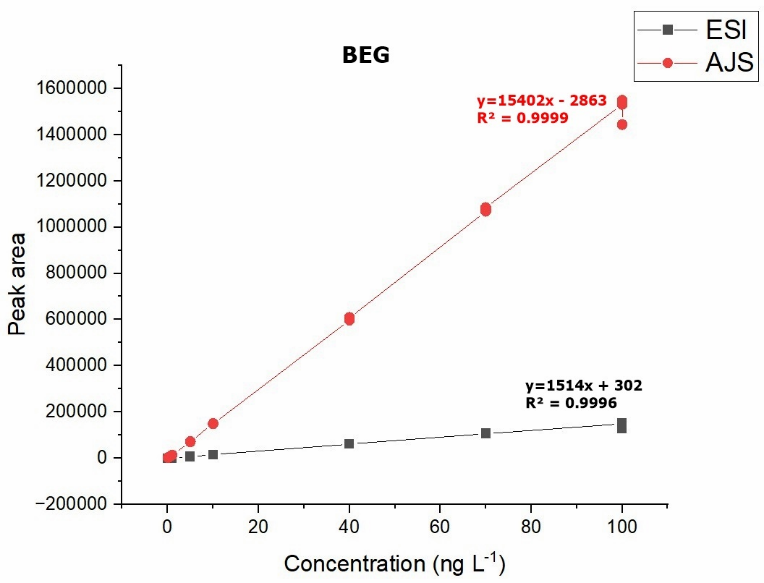


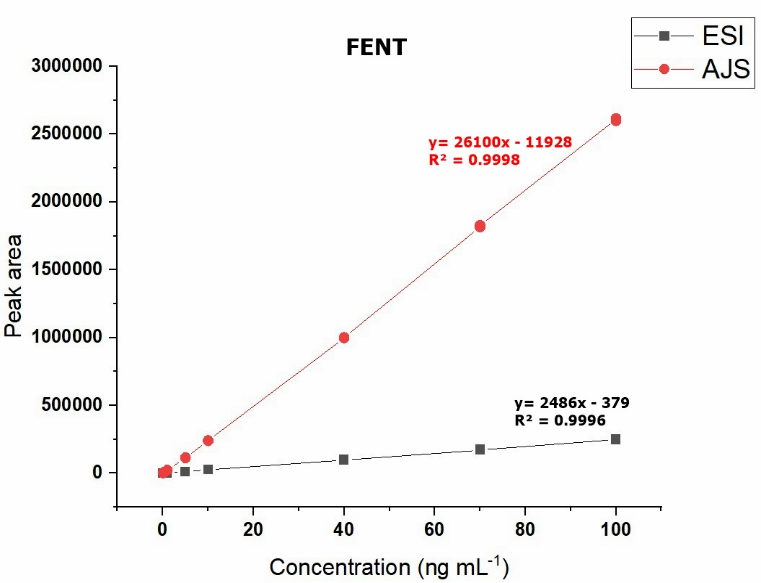


**Fig. S5.** Comparison of slopes of the analytical curves (0.5 to 100 ng mL^-1^) obtained using conventional ESI and AJS electrospray sources. Some examples were shown in the analytical curves of FENT, BEG, COC, and COET.


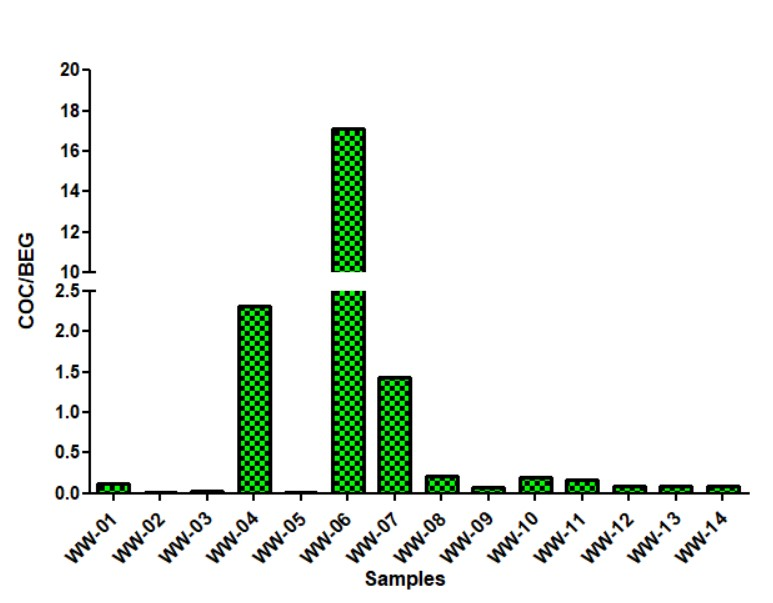


**Fig. S6.** COC/BEG ratios were determined across the wastewater samples investigated. ‘WW’ refers to a specific wastewater sample.

**REFERENCES**

Da Silva, M. C., Oliveira, M. L. G., Augusti, R., & Faria, A. F. (2019). Simultaneous Extraction of Pesticides and Polycyclic Aromatic Hydrocarbons in Brazilian Cachaça Using a Modified QuEChERS Method Followed by Gas Chromatography Coupled to Tandem Mass Spectrometry Quantification. *Journal of Agricultural and Food Chemistry*, *67*(1), 399–405. https://doi.org/10.1021/acs.jafc.8b04682

Johnson, D., Boyes, B., & Orlando, R. (2013). The use of ammonium formate as a mobile-phase modifier for LC-MS/MS analysis of tryptic digests. *Journal of Biomolecular Techniques*, *24*(4), 187–197. https://doi.org/10.7171/jbt.13-2404-005

McCalley, D. V. (2004). Effect of buffer on peak shape of peptides in reversed-phase high performance liquid chromatography. *Journal of Chromatography A*, *1038*(1–2), 77–84. https://doi.org/10.1016/j.chroma.2004.03.038

Nojavan, S., & Yazdanpanah, M. (2017). Micro-solid phase extraction of benzene, toluene, ethylbenzene and xylenes from aqueous solutions using water-insoluble β-cyclodextrin polymer as sorbent. *Journal of Chromatography A*, *1525*, 51–59. https://doi.org/10.1016/j.chroma.2017.10.027

Ribani, M., Collins, C. H., & Bottoli, C. B. G. (2007). Validation of chromatographic methods: Evaluation of detection and quantification limits in the determination of impurities in omeprazole. *Journal of Chromatography A*, *1156*(1-2 SPEC. ISS.), 201–205. https://doi.org/10.1016/j.chroma.2006.12.080

Wu, S., Dong, Y., Lin, B., Shen, X., Xiang, P., & Huang, C. (2022). Sensitive determination of illicit drugs in wastewater using enrichment bag-based liquid-phase microextraction and liquid-chromatography tandem mass spectrometry. *Journal of Chromatography A*, *1661*, 462684. https://doi.org/10.1016/j.chroma.2021.462684
